# What are the symptoms and concerns of young adults living with life-limiting conditions and how well are they captured by patient reported outcome measures? A mixed-methods systematic review and framework synthesis

**DOI:** 10.1177/02692163251405370

**Published:** 2026-01-13

**Authors:** Rachel L. Chambers, India Tunnard, Hannah Scott, Lorna K. Fraser, Katherine E. Sleeman

**Affiliations:** 1 Cicely Saunders Institute of Palliative Care, Policy & Rehabilitation, King’s College London, UK; 2 Department of Women and Children’s Health, School of Life Course and Population Health, Faculty of Life Sciences and Medicine, King’s College London, UK; 3 King’s College Hospital NHS Foundation Trust, London, UK

**Keywords:** young adult, palliative care, symptom, concern, patient reported outcome measure, content validity

## Abstract

**Background:**

Internationally the number of young adults living with life-limiting conditions is increasing. Holistic concerns of this population have not been reviewed. It is unclear whether patient reported outcome measures used in this population capture their symptoms and concerns.

**Aims:**

To: (1) identify and synthesise the symptoms and concerns of young adults (aged 18–39) living with life-limiting conditions; (2) evaluate the content validity of patient reported outcome measures used in this population.

**Design:**

A mixed-methods systematic review and framework synthesis. PROSPERO ID CRD42024565986.

**Data sources:**

MEDLINE, EMBASE, Cochrane, CINAHL, PsycINFO, AMED from inception to 04/07/24.

**Results:**

A total of 16552 articles identified, 100 included. Among these, 34 studies addressed aim 1. Of these 18 were qualitative, 12 mixed methods, 3 quantitative, and 1 used quantitative and qualitative methodologies. 66 quantitative studies addressed aim 2. They used 65 patient reported outcome measures, and 13 questionnaires to capture pre-defined symptoms and concerns. Symptoms and concerns included: physical (fertility and reproductive health, sexual concerns), psychological (embarrassment, suicidal thoughts and body image), social (loss of independence, balancing roles as a parent, missing out on events), spiritual concerns (uncertain future, life on hold), and quality of healthcare concerns (age-specific caring environments, role of partners as caregivers, involvement in decisions). Of the patient reported outcome measures identified as used with young adults, few were holistic.

**Conclusion:**

This review highlights the need for holistic, age-specific person-centred outcome measures for young adults living with life-limiting conditions. We present a conceptual framework of symptoms and concerns that can be used to develop or modify existing patient reported outcome measures for this population.

**What is already known about this topic?**

- Young adults living with life-limiting conditions are a heterogeneous population that is increasing in size and complexity.
- Young adulthood is a time of physical, emotional, social and cognitive development. A greater understanding of the symptoms and concerns of young adults with life-limiting conditions is critical to deliver high quality care.
- The content validity of existing Patient Reported Outcome Measures (PROMs) to assess the symptoms and concerns of young adults remains unclear.

**What this paper adds**

- This review synthesises evidence from 100 primary studies to identify the symptoms and concerns experienced by young adults (aged 18–39) living with a range of life-limiting conditions.
- Symptoms and concerns experienced by young adults include: physical (fertility and reproductive health, sexual concerns), psychological (embarrassment and shame, changes to body image), social (loss of independence and autonomy) and spiritual (life being on hold).
- This review demonstrates that existing patient reported outcome measures do not encompass the symptom and concern domains important to young adults identified in this review.

**Implications for practice, theory or policy**

- There is a need for further research exploring the symptoms and concerns of young adults living with life-limiting conditions. There is an absence of research with young adults experiencing significant developmental delays.
- This review provides a conceptual framework of symptoms and concerns that can be used to develop or modify existing PROMs for young adults living with life-limiting conditions.

## Background

The number of young adults living with life-limiting and life-threatening conditions is increasing.^1^ Life-limiting conditions are defined as ‘individuals living with a condition for which there is no reasonable hope of cure and will cause them to die prematurely’.^2^ Life-threatening conditions are ‘those for which curative treatment may be feasible but can fail, for example, cancer’.^3^ - Within the literature there is no consensus on the definition of ‘young adult’. The definition varies between countries and diagnoses.^4^ In most countries, it is widely accepted that a person becomes an adult at the age of 18. In North America, young adults with cancer are defined by the National Cancer Institute as those aged 15–39 years, a definition derived from the biological characteristics of the types of cancer affecting this age group.^4^

The population of young adults living with life-limiting conditions is heterogeneous, including nearly 400 individual diagnoses.^5,6^ The population comprises two main groups: (1) young adults diagnosed with a life-limiting condition in childhood who survive into young adulthood, where conditions such as congenital and neurological conditions are most prevalent,^5,7^ and (2) young adults diagnosed in young adulthood with conditions such as cancer who do not experience developmental delays. Internationally, the number of young adults living with life-limiting conditions or complex care needs is increasing, but there is a lack of global epidemiological data.^8^ Meeting the concerns of a population that is increasing in size and complexity is challenging.^9^

Young adults living with life-limiting conditions have unique concerns compared to children and adults receiving palliative and end of life care.^10^ They are living with a life-limiting condition at an age of physical, emotional, social and cognitive development. Their concerns may be broader than health, encompassing social concerns such as education and employment.^11,12^ Whilst living with their diagnosis they are often managing their age-specific roles and responsibilities and coping with their ‘truncated life trajectories’ which often affect their aspirations and life goals.^13,14^

A greater understanding of the symptoms and concerns of the young adult population is critical to deliver high quality care. Previous reviews of the symptoms and concerns of young adults living with life-limiting conditions have focussed on: cancer survivors^15,16^; young adults receiving a palliative care intervention^11^; psychosocial concerns^17^; or have assessed the symptoms and concerns of young adults in combination with younger age groups (aged 0–25).^18–20^ To the best of our knowledge, the holistic concerns of young adults living with life-limiting conditions, irrespective of diagnoses, have not been reviewed.

Patient reported outcome measures (PROMs) are standardised questionnaires that can be used to assess patients’ symptoms, quality of life and wellbeing.^19^ Routine use of PROMs in clinical care may facilitate the identification of patient’s concerns, ‘enhance patient/family communication, quality management, evaluation of treatments, and may improve patient outcomes’.^21^

A systematic review of existing PROMs developed, adapted, and validated to assess the health outcomes of young adults (aged 18–25) living with life-limiting conditions found a lack of multi-dimensional PROMs for young adults living with life-limiting conditions, especially for young adults with health conditions other than cancer.^22^ The review also found limited evidence of their psychometric properties, particularly content validity. Further, most of the identified PROMs had been developed without direct input from patients, resulting in questions about their relevance and appropriateness. It therefore remains unclear whether the PROMs used in this population capture outcomes that are important to young adults living with life-limiting conditions.

This systematic review has two parts, corresponding to its two aims. The aims are to: (1) identify and synthesise the symptoms and concerns of young adults (aged 18–39) living with life-limiting conditions; and (2) to evaluate the content validity of PROMs used in this population.

## Methods

### Design

The research paradigm underpinning this mixed-methods systematic review is pragmatism.^23,24^ We placed primary importance on the research question as opposed to the methods used, or the paradigms underlying the methods.^25–28^ Pragmatism represents the different paradigms as ‘two sides of the same coin’. To answer the research questions in our review, it was necessary to synthesise the findings from both quantitative and qualitative studies. Quantitative studies provide information on the presence of symptoms and concerns in the population, while qualitative studies offer rich, contextualised understanding of individuals’ lived experiences. Rather than providing competing metaphysical traits, these aspects provide complementary perspectives^29^ to understand the symptoms and concerns of this population. Thus, the use of mixed methods in this review provides a more complete picture than could be achieved by either a quantitative or qualitative approach alone.

This review was undertaken with a registered protocol (PROSPERO ID: CRD42024565986).

The review is reported in line with the Preferred Reporting Items for Systematic Reviews and Meta-Analysis (PRISMA) guidelines (Supplemental File 1).^30^

### Searches

We searched MEDLINE; EMBASE; CINAHL; PsycInfo; AMED via Ovid and Cochrane Library from inception to 04/07/2024. Searches were supplemented by hand-searching reference lists of systematic reviews and included articles.

#### Search strategy

The search was developed in MEDLINE using text words and subject headings with input and support from a librarian at King’s College London with expertise in systematic searches in medical research databases. The search strategy was adapted from previous systematic reviews.^18,31^ The search strategy used MeSH terms and synonyms for the following search concepts: ‘young adult’, ‘life-limiting and life-threatening illness’ and ‘symptoms and concerns’ (Supplemental file 2). The search concepts for ‘young adults’ and ‘life-limiting and life-threatening illness’ were adapted from Knighting et al. ’s systematic review of respite care and short breaks for young adults aged 18–40 years with complex healthcare needs.^31^ The search concepts for ‘symptoms and concerns’ were adapted from Namisango et al’s systematic review on symptoms and concerns among children and young people with life-limiting and life-threatening conditions.^18^

No date or language limits were applied.

#### Eligibility criteria

Primary full-text peer-reviewed research studies, reporting the symptoms and concerns of young adults living with life-limiting conditions were included. Within the literature there is no consensus on the definition of ‘young adult’.^4^ In most countries, a person becomes an adult at the age of 18. Within cancer young adults include those aged 15–39 years.^4^ For this review, we define young adults as those aged 18–39.

Detailed inclusion and exclusion criteria are shown in Table 1.

**Table 1.** Inclusion/exclusion criteria.

Concept	Inclusion criteria	Exclusion criteria
Population	Study population that includes young adults (aged 18–39) living with a life-limiting condition (defined as individuals living with a condition for which there is no reasonable hope of cure and will cause them to die prematurely) and life-threatening condition (for which curative treatment may be feasible but can fail).^2,3^ Studies of a mixed population will be considered if the mean/median age falls within the age range 18–39 years. If the mean/median age is not reported, studies of a mixed population will be considered for inclusion if the majority (⩾50%) of participants are aged 18–39 years. Studies of a mixed population will be included where the results are stratified by age.	Study population solely comprising children or adults (i.e., aged < 18 or ⩾40 years). Mean or median age is not reported or does not fall within the range of 18–39 years. Or the percentage of young adults aged 18–39 years is <50%. Or studies of a mixed population that do not stratify their results by age. Cancer survivors.
Context	Patients in all settings, in any country.	No study will be excluded based on the context.
Outcomes	Symptoms and concerns of young adults (aged 18–39 years) living with life-limiting conditions. Patient and proxy (e.g. family and healthcare professional) reported symptoms and concerns will be included.	Focus is on the concerns of the family only (e.g. excluding the young adult). Focus is on the concerns of the healthcare professional only (e.g. excluding the young adult).
Study type	Original, full-text peer-reviewed research studies of all types. There are no publication date limits.	Case studies, descriptive, theoretical, or clinical opinion articles, theses, books, editorials, conference abstracts and protocols. Non-peer reviewed papers. Scoping and systematic reviews (reference lists will be hand-searched). Full text not available.

### Study selection

The search results were imported into Covidence (www.covidence.org). Covidence was used to manage the large number of references, remove duplicates and to manage the screening and selection of papers.

The first author (RLC) independently screened titles and abstracts against the inclusion criteria. A subset (20%) of papers were randomly selected for double-screening by a second reviewer (HS). There was 97% consensus on papers to be included, excluded and where there was uncertainty. Uncertainties and conflicts were resolved through discussion and consultation with LKF and KES.^32^

At full text stage, RLC and HS independently screened all full texts against the inclusion criteria. Conflicts were resolved by discussion. There was 96% consensus on papers to be included, excluded and where there was uncertainty. Uncertainties and conflicts were resolved through discussion and consultation with LKF and KES. Reasons for exclusion at full text were recorded.^32^

### Data extraction and synthesis

RLC independently extracted data from each study and entered the data into a Microsoft Excel spreadsheet using a piloted data extraction template. We extracted data on study characteristics, participant characteristics, symptoms and concerns (aim 1), the name of PROMs (where relevant) and the content of items included in each PROM (aim 2). This information was extracted to later map the symptoms and concerns onto existing PROMs. We extracted direct quotes on symptoms and concerns from qualitative studies where possible.

A subset (20%) of the extracted data was checked by a second reviewer (IT).

For aim 1 a convergent integrated approach was used to synthesise the symptoms and concerns identified by quantitative and qualitative papers.^33^

In line with previous mixed-methods reviews,^32^ numerical and statistical findings from quantitative papers were converted into descriptive summaries. For qualitative data, ‘meaning units’ (comprehensible segments of text which contain one idea or piece of information) were identified and coded drawing on author-reported results and participant quotes.

The study findings were analysed and combined using a framework approach.^34–36^ The World Health Organisation’s (WHO) definition of Palliative Care (physical, psychological, social and spiritual) was selected a priori as the framework.^35–37^

Within excel, we labelled columns separately for each of the themes (physical, psychological, social and spiritual). We used these themes to deductively code the symptoms and concerns identified. We allowed for symptoms and concerns that did not fit into these four themes by creating a separate column labelled ‘other’.^18^ This approach allowed for the framework to be expanded and added to as needed.^38^ This was completed by one reviewer (RLC) with regular input from the wider research team for sense checking and validation.

Data were then synthesised within the five themes.^39^ Colour coding was used to group similar symptoms and concerns. To interrogate the data, we developed matrices to compare symptoms and concerns by method of data collection (e.g. clinical record reviews, interviews, blog posts etc.) and diagnostic groups. Data from quantitative and qualitative studies were grouped together into subthemes based on similarity in meaning. Reference was made to the classifications used in Namisango et al’s review of symptoms and concerns of children and young people living with life-limiting and life-threatening conditions,^18^ which used a similar method. Subthemes were verified with the primary data sources to ensure accuracy. Themes and subthemes were finalised through discussions as a research team.

#### Assessment of methodological quality

To assess the methodological quality of studies we used the Hawker et al. checklist for reviewing disparate data systematically.^40,41^ The checklist comprises nine questions, assessing the abstract, background, methodology, sampling, data analysis, ethics, results, transferability and implications. Each element is rated on a four-point scale ranging from ‘good’ (4), ‘fair’ (3), ‘poor’ (2) to ‘very poor’ (1). The total score can range from 9 to 36. In line with previous reviews, studies scoring ⩽18 were rated as ‘poor’, 19–27 were rated as ‘fair’ and >27 as ‘good’.^41,42^

One reviewer (RLC) independently assessed the methodological quality of included studies. A second reviewer (IT) independently quality appraised 20% of included papers. Scores were compared for consistency. Conflicts were resolved through discussion and consultation with the wider research team. A prior decision was made not to exclude studies for methodological reasons, instead we chose to report the quality assessments to describe the quality of research evidence in this area.^43,44^

For aim 2, we constructed a table of patient reported outcome measures for young adults living with life-limiting conditions by extracting validated PROMs from Chambers et al. systematic review of PROMs developed, adapted or validated for the young adult population.^22^ This was supplemented by PROMs used by studies identified in this review.

The themes and subthemes of symptoms and concerns from aim 1 were used as analytical codes that were mapped onto the content of items from existing PROMs, to examine the degree of content coverage. We calculated the percentage of content coverage for each PROM at subdomain level. A high or strong match was identified when the content of the PROM represented a large majority (greater than 80%) of symptom and concern themes identified by this review.^45^ A moderate match was identified when the content captured 30%–79%. A low or weak match was identified when the content captured less than 30%.^46^

## Results

**Figure 1.**
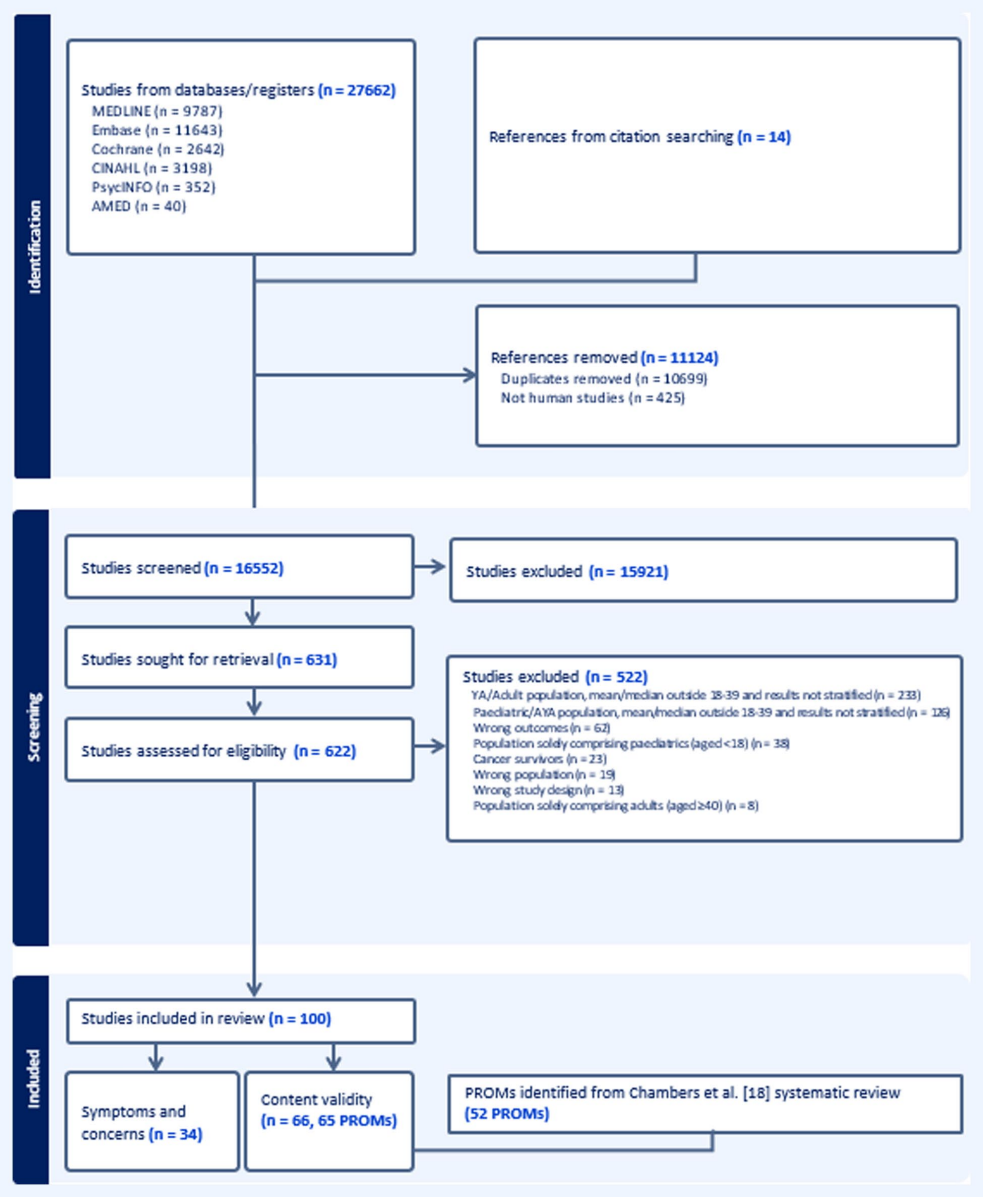
PRISMA flow chart detailing search results and reasons for study exclusion.

After de-duplication, 16,552 titles and abstracts were screened, 622 full-text papers were reviewed. One hundred papers met the inclusion criteria (see Figure 1).

### Aim 1: Identification of symptoms and concerns

#### Study characteristics

Thirty-four studies were included in the first part of the study (aim 1; Table 2). The 34 papers were published between 1988 and 2024. Of these, 18 were qualitative, using methods such as focus groups, semi-structured interviews and content analysis of blog posts to identify symptoms and concerns. Twelve studies were mixed methods. Some used questionnaires alongside semi-structured interviews and focus group discussions to identify symptoms and concerns. Three studies were quantitative; one study asked patient to self-report symptoms using an app, another extracted self-reported symptoms from clinical records, and the third used a consensus method to identify the most important symptoms and concerns. One study used both quantitative and qualitative methodologies: they translated a patient reported outcome measure, followed by cognitive interviews and a feasibility study.

**Table 2.** Characteristics of included studies (*n* = 34).

Author, year	Country	Study design	Outcome measure	Aim(s)	Setting	Sample size	Age (mean, SD)	Gender	Race/ethnicity	Hawker et al.′s quality score^40^
Cancer										
Alander et al. 2021^47^	Sweden	Qualitative; narrative analysis	Not applicable	To explore the lived experience of young adults diagnosed with cancer and to increase our understanding of how to help them with their caring needs	Online blog	8	Range 20–29	Female 100%	Not reported	30; Good
Ameringer et al. 2015^48^	United States	Mixed methods	Memorial symptom assessment scale	(1) To explore symptoms and symptom clusters from the perspectives of AYAs using the Computerised Symptom Capture Tool (C-SCAT) (2) To describe symptoms, symptom clusters, and priority symptoms within clusters (3) To describe the perceived causes of individual symptoms (4) To examine the complexity of symptoms and symptom clusters	Medical centres	72	18.5 (4.2)	Female 43.1% Male 56.9%	White/Non-Hispanic 79.2% Black/African American 9.7% Hispanic 5.6% Asian/Pacific Islander 4.2% Other 1.4%	33; Good
Avutu et al. 2022^49^	United States	Qualitative; focus groups	Not applicable	(1) To explore the unique experiences of cancer care as an AYA, including self-identified physical, social, and emotional concerns (2) To identify AYA-specific information needs and communication preferences, including the appropriateness, timing, and depth of information delivered (3) To provide recommendations and direct for service provision and delivery, and accommodations for AYAs throughout their cancer experience	Medical centres	25	26.8 (5.9) Range 16–39	Female 54.2% Male 41.7% Genderqueer/Gender non-binary 4.2%	Caucasian/White 70.8% Black/African American 12.5% Asian 8.3% Prefer not to answer 8.3%	30; Good
Barakat et al. 2016^50^	United States	Mixed methods	Not applicable	To better understand psychosocial care needs and programme preferences to inform development of more easily accessible and effective AYA psychosocial programmes	Cancer centre	111	17.37 (2.73) Range 12–25	Female 52.4%	Not reported	32; Good
Choi et al. 2022^51^	United States Australia Canada England South Africa	Mixed methods	Not applicable	To identify and compare the unmet needs of adolescents and young adults (AYAs) with cancer by age and gender	Online blog	100	29.22 (6.71) Range 18–39	Female 50.0% Male 50.0%	Not reported	33; Good
David et al. 2012^52^	Wales	Qualitative: focus groups	Not applicable	To facilitate a small scale qualitative, focus group for TYA patients who had received radiotherapy treatment within Wales to identify any issues related to psychological support	Cancer centre	4	Range 18–24	Female 75.0% Male 25.0%	Not reported	28; Good
Erickson et al. 2019^53^	United States	Mixed methods	PROMIS self-efficacy for managing symptoms scale	To examine the effects of a heuristic symptom assessment tool, the Computerised Symptom Capture Assessment Tool (C-SCAT) on AYA’s self-efficacy for symptom management, their self-regulation abilities related to symptoms, ad negotiated collaboration, as operationalised by AYA’s perceptions of communication with their providers about symptoms	Medical centres	85	20.9 (4.92) Range 15–29	Female 46% Male 54%	White 72% Other or more than one race 13% African-American 12% Native American/Alaska Native 2% Asian 1%	33; Good
Hirayama et al. 2023^54^	Japan	Quantitative and qualitative; validation study	Japanese version of the distress thermometer and problem-list	(1) To develop a screening tool for determining distress and supportive care needs of adolescent and young adult cancer patients (AYAs) based on the NCCN’s Distress Thermometer and Problem List (DTPL) (2) To evaluate its feasibility, discriminant validity, and test–reliability	Hospital	40	Development phase 26.7 (7.0) Range 15–39 Feasibility phase: 25.7 (8.1) Range 15–39	Development phase Female 50% Male 50% Feasibility phase Female 55.7% Male 44.3%	Not reported	34; Good
Kohi et al. 2019^55^	Tanzania	Qualitative; focus groups	Not applicable	To explore cancer-related concerns and needs of care and support among young adults and children who are receiving cancer treatment in Dar es Salaam, Tanzania	Hospital	8	21.0 (2.6)	Female 50% Male 50%	Not reported	30; Good
Lea et al. 2020^56^	United Kingdom	Mixed methods	Not applicable	To understand the needs of young people with cancer, how these needs are currently being met, and how best to provide information and support at the end of active treatment	Cancer settings	Interviews 11	Range 19–26	Female 73%	Not reported	27; Good
Lidington et al. 2021^57^	United Kingdom	Qualitative: interviews and focus groups	Not applicable	(1) To explore the specific experiences of young adults diagnosed with cancer in the UK context (2) To describe the age-specific psychosocial impact and practical challenges of cancer and its treatment	Hospital	65	33.6 Range 25–42	Female 60.0% Male 40.0%	White 74.6% Asian/Asian British 16.4% Black/African/Caribbean/Black British 3.0% Mixed/Multiple ethnic groups 3.0%	35; Good
Linder et al. 2019^58^	United States	Mixed methods	Memorial symptom assessment scale	(1) To describe the frequency and characteristics of priority symptoms (2) To explore reasons for a symptom’s designation as a priority symptom, perceived causes of priority symptoms, and strategies AYAs use to manage priority symptoms	Medical centres	86	21 (5.0) Range 15–29	Female 47.7% Male 52.3%	White 70.9% Other/more than one race 14% African-American 11.6% Native American/Alaska Native 2.3% Asian 1.2%	29; Good
Locatelli et al. 2023^59^	Denmark	Quantitative; cross-sectional	Not applicable	(1) To investigate symptom patterns in young adults with cancer using a smartphone-based app (2) To explore symptom frequency and severity, cluster patients based on their symptom severity, investigate the co-occurrence of severe symptoms, and explore the relationship between symptoms and activities	Hospital	161	25.49 (4.68)	Female 75%	Not reported	27; Good
Odh et al. 2016^60^	Sweden	Qualitative; content analysis	Not applicable	To elucidate the theoretical framework of Yalom and his four ‘givens’ which were expressed in blogs written by young adults living with various cancer diagnosis in Sweden	Online blog	13	Not reported	Not reported	Not reported	29; Good
Park et al. 2023^61^	South Korea	Mixed methods	Not applicable	To evaluate the care needs of AYAs with cancer considering the cultural background and health care system for cancer treatment in South Korea	Cancer centre	Survey 77 Focus groups 10	Survey 21.1 (7.4) Range 15–39 Focus groups Range 15–39 15–18 (50%) 19–39 (50%)	Female 46.8% Male 53.2%	Not reported	31; Good
Patterson et al. 2012^62^	Australia	Qualitative; focus groups and interviews	Not applicable	To inform our understanding of the needs of emerging adults with a diagnosis of cancer from a developmental perspective that appreciates the key transitional tasks of emerging adulthood	Hospital	14	22 Range 20–25	Female 57.1% Male 42.9%	Not reported	29; Good
Rana et al. 2017^63^	Canada	Qualitative; focus groups and interviews	Not applicable	To gather and analyse the physical, psycho-social and informational needs of YWBC at a regional cancer centre	Cancer centre	16	37 Range 30–40	Female 100%	Not reported	29; Good
Ruddy et al. 2013^64^	United States	Qualitative; focus groups	Not applicable	To elucidate which issues are most disturbing to this breast cancer patients and which might be amenable to intervention	Cancer centre	36	37.8 (4.7) Range 26–44	Female 100%	White non-Hispanic 94%	30; Good
Ruddy et al. 2015^65^	United States Canada	Qualitative; interviews	Not applicable	To elucidate which issues are most disturbing to this breast cancer patients and which might be amenable to intervention	Hospital	20	35.9 (5.0) Range 26–42	Female 100%	White non-Hispanic 50% Hispanic 25% Black 15% Other (native Hawaiian, Caribbean) 10%	31; Good
Simon et al. 2023^66^	United States	Mixed methods	Not applicable	To assess the most salient health care needs of adolescents and young adults (AYAs) who undergo cancer treatment	Medical centres	10	21 Range 16–29	Female 40% Male 60%	White 60% Hispanic 20% Asian/Pacific Islander 10% Multiracial 10%	32; Good
Takeuchi et al. 2019^67^	Japan	Qualitative; medical records content analysis	Not applicable	To identify support needs among young cancer patients regarding fertility-related issues to describe multidimensional support provided by nonphysician health care providers	Hospital	51	0–19 (2.0%) 20–29 (11.8%) 30–39 (54.9%) 40+ (31.4%)	Female 78.4% Male 21.6%	Not reported	31; Good
Tan et al. 2024^68^	United States	Mixed methods	Reproductive concerns after cancer scale	To explore and differentiate the reproductive concerns of AYAs	Cancer centres	27	19.1 (3.7)	Female 37.0% Male 63.0%	White 81.5% Asian 18.5%	33; Good
Spina bifida										
Starowicz et al. 2021^69^	Canada	Quantitative; retrospective cohort chart review	Not applicable	To identify the most common health concerns among TranLC patients with SB at initial clinical consultation	Healthcare centre	94	29.04 (13.8) Range 13–77	Female 70.2% Male 29.8%	Not reported	31; Good
Cystic fibrosis										
Brissette et al. 1988^70^	Canada	Mixed methods	Not reported	To understand the concerns of cystic fibrosis severely ill young adults	Home	12	Range 17–27	Not reported	Not reported	17; Poor
Dellon et al. 2010^71^	United States	Qualitative; interviews	Not applicable	To describe symptom prevalence, symptom management, and frequency of use of disease-specific treatments for patients dying from complications of CF	Not reported	27	Mean age at death 24 Range 8–47	Female 67%	Not reported	31; Good
Hailey et al. 2019^72^	United States	Qualitative; interviews	Cystic fibrosis questionnaire-revised	(1) To describe the parenting and reproductive health concerns of parents and non-parents with cystic fibrosis, including men and women (2) To identify the psychosocial and educational needs of individuals with cystic fibrosis who are considering parenthood or are parents	Hospital	20	Median 28.5 Range 22–46	Female 50% Male 50%	Caucasian 90% African American 10%	33; Good
Congenital heart disease										
Chen et al. 2017^73^	Taiwan	Quantitative; delphi survey	Not applicable	This study systematically identified the healthcare needs of adolescents with CHD transitioning into young adults by collecting the perspectives of patients, parents, and healthcare providers	Clinic	29	Median 20.8 Range 15–24	Female 41.4% Male 58.6%	Not reported	33; Good
HIV										
Jameson et al. 2008^74^	South Africa	Qualitative; interviews	Not applicable	To investigate the palliative care needs of patients with stage 3 and 4 HIV infection in Settlers Hospital, Grahamstown	Hospital	50	36	Female 70%	Not reported	22; Fair
Selman et al. 2013^75^	Kenya Uganda	Qualitative; interviews	Not applicable	(1) To describe the palliative care needs of HIV outpatients and the management of multidimensional problems by HIV outpatient services in Kenya and Uganda (2) To inform the provision of HIV care and support in sub-Saharan Africa	Outpatient	83	36 Range 18–61	Female 58%	Not reported	35; Good
Uwimana et al. 2007^76^	Rwanda	Mixed methods	Not applicable	(1) To identify palliative care needs of people living with HIV/AIDS in selected areas in Rwanda (2) To determine if these needs were met or unmet	Hospitals Health centres Day centres	250	38	Female 71% Male 29%	Not reported	30; Good
Chronic kidney disease										
Kubiak et al. 2023^77^	Germany	Qualitative; interviews	Not applicable	To identify the point of view of adolescent and young adult patients as well as caregivers of younger children with advanced CKD	Hospital	5	Median 19 Range 16–22	Female 60% Male 40%	Not reported	31; Good
Duchenne muscular dystrophy										
Rosero et al. 2024^78^	United States	Mixed methods	Not applicable	(1) To determine the prevalence and relative importance of symptoms experienced by individuals with Duchenne Muscular Dystrophy (2) To identify factors associated with a higher disease burden	Duchenne Muscular Dystrophy clinic	Interviews 13 Survey 87	Phase 2 25.74 (13.31) Range 10–74	Phase 2 Female 6.90% Male 91.95% Other 1.15%	Phase 2 White 85.06% Hispanic/Latino 13.79% Other 6.90% Asian 5.75% Black/African-American 2.30%	30; Good
Barth syndrome										
Gwaltney et al. 2021^79^	United States	Qualitative; validation study, interviews	Barth syndrome-symptom assessment	(1) To develop symptom-focussed patient-reported outcome (PRO) measures for use in clinical studies with adolescents and adults with Barth Syndrome (2) To describe the initial development of the BTHS-SA and evidence supporting its content validity as defined in regulatory guidance documents and expert guidelines (i.e. that the BTHS-SA targets relevant and important symptoms of BTHS and is both understood and used as intended by respondents	Not reported	Concept elicitation: 15 Cognitive debriefing: 12	Concept elicitation: 22.9 (5.8) Range 16–34 Cognitive debriefing: 22.9 (6.1) Range 16–35	Not reported	Cognitive elicitation: White 93.3% Black or African American 6.7% Cognitive debriefing: White 100%	32; Good
Sickle cell disease										
Matthie et al. 2016^80^	United States	Qualitative; interviews	Not applicable	To describe the perceptions of young adults with sickle cell disease concerning their disease experience	Clinics	29	25.8 (4.8) Range 20–31	Female 79.3%	African-American 100%	34; Good

Twenty-seven were from high-income countries: 13 from the USA, 3 each from Canada and the UK, 2 each from Japan and Sweden, and 1 each from Australia, Denmark, Germany and South Korea. Four were from low- and middle- income countries, one each from: Tanzania, Rwanda, South Africa and Taiwan. Three were multi-national (see Table 2).

Most studies included patients living with cancer (*n* = 22). Other studies recruited patients living with cystic fibrosis (*n* = 3), HIV/AIDS (*n* = 3), sickle cell disease (*n* = 1), spina bifida (*n* = 1), Duchenne muscular dystrophy (*n* = 1), congenital heart disease (*n* = 1), Barth syndrome (*n* = 1), and chronic kidney disease (*n* = 1).

#### Quality of included studies

Of the 34 studies, Hawker et al’s quality scores ranged from 17 to 34 out of a possible score of 40. The range of scores were 27–33 (fair-good) for quantitative studies, 17–33 (fair-good) for mixed method studies, 22–35 (fair-good) for qualitative studies and 34 (good) for a study that used quantitative and qualitative methodologies (see Table 2). Studies often scored lower in the following domains: ethics, sampling and implications of findings. Most stated that ethical approval was obtained but did not mention issues of confidentiality, sensitivity and consent, or researcher reflexivity for qualitative studies. Some authors did not adequately describe how they recruited participants and provided limited information on participant characteristics (e.g. age, gender, race/ethnicity).

**Figure 2.**
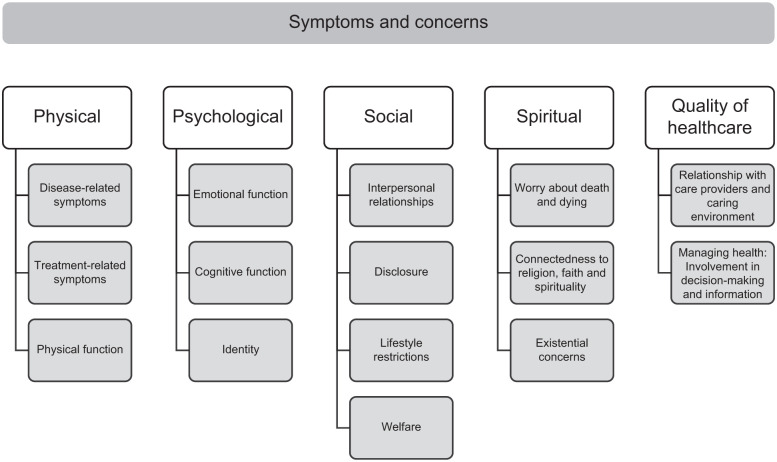
Synthesised findings of the symptoms and concerns of young adults living with life-limiting conditions.

Within the 5 themes (physical, psychological, social, spiritual and quality healthcare), 15 subthemes were identified (see Figure 2). The synthesis of results by themes and subthemes is summarised below and in detail in Supplemental File 4.

##### Physical

Disease-related symptoms included pain fatigue and breathlessness.

*I just struggle day to day just to make it and not have pain* (Young adult living with Sickle Cell Disease)^80^ (p. 1445).

Treatment-related concerns included fertility and reproductive health. These concerns were reported by young adults living with cancer, spina bifida, cystic fibrosis and HIV. Other treatment related concerns for young adults living with cancer included hair loss, premature menopause, and hot flushes. HIV patients reported side-effects of antiretroviral therapy including loss of appetite, vomiting and dizziness.

*The fertility issue is a big issue, and to have to make a decision on that, in that head space, I think, is really challenging. There’s so much pressure. ‘Oh, my God, I have to decide if I want to have kids in 5 years, right this minute!’ Which is a really weird place to be in where you’re thinking about your own mortality*. (Young adult living with cancer)^64^ (p. 155).

Some symptoms including nausea and constipation could be classified as both disease-related and treatment-related.

Young adults expressed the impact of their disease on their physical function including their mobility, and their ability to perform personal care tasks such as bathing.

*They gave me a zimmer frame. I actually looked at it and said, no, I’m not using that. . . I’m 37 years old. I’m not 78 years old. I didn’t want to do it. I’m very proud and private and I didn’t want to be seen as old and frail. . .* (Young adult living with cancer)^57^ (p. 5).

Sexual concerns were also included within physical function and were reported by young adults living with cancer, sickle cell disease, spina bifida and cystic fibrosis. These concerns included changes to sexual function (such as erectile dysfunction) and loss of libido.

*The one thing that was never addressed . . . is how all of this affects or can affect a woman’s sex drive. . .* (Young adult living with cancer)^63^ (p. 6).

##### Psychological

Across all diagnoses, young adults highlighted emotional concerns, describing the negative effect of their illness experience and the impact on their mood. Mood-related symptoms included depression, anxiety, and feeling fearful, particularly around the trajectory of their disease. Individuals living with cancer reported fears of relapse, recurrence and of their cancer spreading. Patients living with chronic kidney disease were similarly fearful of recurrence, whilst patients living with cystic fibrosis reported fears of disease progression.

Young adults expressed feelings of embarrassment and shame because of their illness. Further, suicidal thoughts and ideation were reported by a minority of young adults living with cancer and cystic fibrosis.

*I wonder if I’m ever going to be free from the dark thoughts. They are eating me up from the inside out.* (Young adult living with cancer)^60^ (p. 57).

Cognitive concerns were reported across diagnoses. These included attention difficulties, memory problems, confusion, difficulties concentrating, and communication difficulties.

Young adults described changes to their body image and their perception of self because of their illness. Young adults living with cancer described an altered sense of self due to changes in their physical appearance. In one qualitative study, young adults described acquiring an illness identity.

*. . .I want to be me and I haven’t been able to be me since being diagnosed and I will never be me again* (Young adult living with cancer)^57^ (p. 5).

##### Social

Young adults wanted interpersonal support from their romantic partners, friends and family. Young adults were concerned about disclosing their diagnosis to others for fear of being treated differently and wanting to protect their loved ones from the emotional impact of their disease, particularly their children:

*. . .having that conversation I have yet to have with my children because they are just too young explaining to them what CF is, how it affects me, and trying to educate them on what’s going on with me and not to scare them, I don’t want them thinking I am going to die tomorrow.* (Young adult living with cystic fibrosis)^72^ (p. 1230).

Young adults expressed difficulties balancing their role as a parent, with their new identity as a patient.

*Finding the balance between keeping myself as healthy as possible while keeping our family a happy working family unit . . . that’s always going to be a bit of a struggle* (Young adult living with cystic fibrosis)^72^ (p. 1228).

Some young adults described being different to other people living with cancer, because of their age, and wanted to talk to others who shared similar experiences in the form of peer support groups. This was predominately reported by young adults living with cancer but was also reported by some young adults living with congenital heart disease and spina bifida.

*A lot of times when you go to treatments or appointments, you’re the only young person there. Everyone’s older, so you kind of—feel like an outsider I guess. It’s nice to connect with other people going through the same thing* (Young adult living with cancer)^49^ (p. 8).

Some young adults limited their recreational activities (sports, social activities) because of their illness. Unlike their peers, young adults often missed out on important life events, typical for their developmental age, such as going to school or ‘doing crazy teenager things’^62^ (p. 36):

*All your friends are doing ‘crazy teenager’ things, and you have to deal with the possibility of dying.* (Young adult living with cancer)^62^ (p. 36).

Some also experienced disruptions to their work. Prior to their diagnosis, young adults prioritised their career, but often, their diagnosis forced them to take a period of absence.

*I lost a lot of jobs behind being sick and in pain and lot of jobs were challenging because of the pains.* (Young adult living with Sickle Cell Disease)^80^ (p. 1446).

A loss of independence and autonomy due to their diagnosis was also described as being difficult to accept. Some compared their life pre- and post-diagnosis, emphasising a sense of loss over the life they used to have:

*I was living away from home, working when I was diagnosed. When I was diagnosed, I relocated back home and was living with my parents then after being away for six years. And I felt like it was challenging. My parents were great, and they attended all of my meetings and came to the hospital with me every day, but it was difficult going from being independent to then depending on my parents again.* (Young adult with cancer)^49^ (p. 8).

##### Spiritual

Worries about death and dying were reported by young adults living with cancer, congenital heart disease and sickle cell disease. Specifically, young adults living with cancer reported thoughts about dying, fearing death, and coming to terms with the possibility of their own mortality.

*I’m afraid. So incredibly afraid of dying. I’m afraid of the anxiety you would feel before. Or if you knew there was nothing more to do.* (Young adult living with cancer)^60^ (p. 58).

Some young adults used their spirituality and connection with faith and religion to cope with their diagnosis and expressed a desire for inner peace. Patients felt comforted by this existential support:

*Religion plays a huge role. My family . . .religion has always been an essential thing in our family and my relationship with the Lord and just involvement in church, it played a big role for me and as far as my sickle cell and, number one, just helping me to stay positive to realize that, OK, yeah, I might have this, but there is obviously a purpose or reason for it and even though I don’t understand it, that I’m going to be OK and just helping me to stay positive.* (Young adult living with Sickle Cell Disease)^80^ (p. 1445).

Young adults expressed existential concefrns such as uncertainty about the future and feeling like their life is on hold because of their illness. Others shared that their illness led them to find meaning and purpose in their lives, and some accepted their disease.

*Yeah, I would love to not have sickle cell no more, but I’ve grown to accept it. It’s a part of me. . .It’s not who I am, but this is what I have to live with* (Young adult living with Sickle Cell Disease)^80^ (p. 1445).

##### Quality of healthcare

Some symptoms and concerns identified could not be categorised into the themes of palliative care. These concerns centred around the overarching theme of quality of healthcare.

Young adults wanted an honest and trustworthy relationship with their healthcare professionals. They wanted to be treated as an adult, not as a child:

*Doctors don’t really know how to talk to you and approach you, they treat you like a child.* (Young adult living with cancer)^62^ (p. 35).

In one study,^49^ young adults wanted their healthcare professionals to acknowledge the role of their partners as primary caregivers, and to not automatically assume that this role was assigned to their parents. Keeping this information up to date was seen as important:

*So one of my providers were used to deferring to the mother and not recognizing that the person who lived with me and built a life with me was probably a little bit more attuned to how I was behaving, even though my mother had been my primary caregiver [in] the past [ . . . ] nobody had talked to me before I started treatment again about updating my proxy, even though they knew very well that my social situation had changed and that my partner would be my primary caregiver. They didn’t think to tell me to update my proxy and so I didn’t* (Young adult living with cancer)^49^ (p. 11).

Young adults shared that they were sometimes cared for in paediatric or adult settings where they felt uncomfortable and out of place:

*When I was hospitalized for chemotherapy, I was always the youngest in the hospital room. I felt uncomfortable when other patients and their families looked at me like ‘why is that child here?’* (Young adult living with cancer)^61^ (p. 404).

Young adults wanted information about their disease and diagnosis. They specifically wanted information about the causes of symptoms, treatment options, and information about their transition from child to adult services. Other information needed included birth control methods, family planning, the impact of pregnancy on their health, fertility preservation and treatment, and the heritability of their own disease and how it may impact their children.

Some young adults living with cancer expressed a desire to be involved in decision-making, to advocate for themselves and their own preferences for care.

### Aim 2: Content validity of patient reported outcome measures

This review identified 66 quantitative studies that were included in the second part of the study (aim 2). Of these 53^10,81–133^ used 65 patient reported outcome measures and 13 used questionnaires to capture pre-defined symptoms and concerns^59,134–145^ (see Supplemental File 3). In addition to the 65 PROMs identified, we included an additional 52 PROMs from Chambers et al. systematic review of PROMs developed, adapted or validated for the young adult population.^22^

Content coverage of all 117 PROMs was mapped against the themes and subthemes identified in Aim 1. The results are presented in Supplemental File 5. Of the 117 PROMs, 60 had less than 30% content coverage, 53 had coverage of 30%–79% of the subthemes, and 4 PROMs had at least 80% content coverage.

Eighty-one PROMs (69.2%) assessed physical symptoms and concerns, often disease and treatment-related symptoms. Of these, one-third (*n* = 27, 33.3%) assessed concerns surrounding physical function. Over three quarters of PROMs (*n* = 91, 77.8%) assessed psychological concerns, mostly assessing emotional and cognitive function. Of these, a small number of PROMs (*n* = 16, 17.6%) assessed concerns around identity. 82 PROMs (70.1%) assessed social concerns. Most focussed on interpersonal relationships and lifestyle restrictions. Of these, few captured welfare concerns (e.g. financial or housing concerns; *n* = 15, 18.3%). Some PROMs assessed spiritual concerns (*n* = 40, 34.2%). Most of these PROMs captured existential concerns (e.g. uncertainty about the future, life being on hold). Twenty-three PROMs (19.7%) assessed quality of healthcare.

## Discussion

### Main findings of the study

This review identified a large volume of literature describing the symptoms and concerns of young adults (aged 18–39) living with life-limiting conditions. Results show the symptoms and concerns of this population are multidimensional, spanning physical, psychological, social, spiritual and quality of care domains. These findings align with previous research which shows that the concerns of individuals living with life-limiting conditions are holistic.^11,18^ We identified a range of concerns that were specific to young adults and may be related to their developmental stage.^11^ We identified 117 patient reported outcome measures being used with the young adult population, however even the most comprehensive tools do not capture all domains important to young adults living with life-limiting conditions.

### What this study adds

Our review identified many studies reporting the symptoms and concerns of young adults living with cancer. Many non-cancerous conditions were under-represented in this review, especially complex neurodisability, demonstrating an absence of research. Whilst it may not be possible to have representation across all diagnoses, there is a need for research exploring the symptoms and concerns of young adults living with a range of conditions to ensure their symptoms and concerns are represented,^146^ particularly those experiencing significant developmental delays.

Previous research has shown that care providers should encourage young adults to develop autonomy through the care they provide, and involve their young adult patients in decisions about their care,^147^ a concern that was identified by this review. We also found that young adults wanted a good relationship with healthcare professionals to allow for direct and honest communication. This finding aligns with previous research which highlights changes in social and professional relationships across the illness trajectory, and how these can be acknowledged and managed by healthcare professionals.^148,149^

We identified four PROMs that had at least 80% coverage, but the number of items included in each measure ranged from 42 to 139. In PROM development there is a need for balance between comprehensiveness and feasibility and acceptability. Long measures may burden patients with questionnaire fatigue, particularly for individuals approaching the end of life.^150^ The European Association for Palliative Care (EAPC) task force on outcome measurement has called for researchers and clinicians to consider patient burden when selecting measures for use and to select sufficiently brief measures where possible.^151^ There are existing holistic measures that have been developed to assess the symptoms and concerns of adults (e.g. Integrated Palliative care Outcome Scale^152^) and children (Children’s Palliative care Outcome Scale^153^) living with life-limiting conditions. These measures were not identified by our searches due to our inclusion criteria for age. But these measures are sufficiently brief (⩽17 items) and, if modified or adapted, may better capture the symptoms and concerns of young adults living with life-limiting conditions.

Most PROMs identified assessed physical, psychological and social symptoms and concerns, with few assessing spiritual concerns and quality of healthcare. This finding may be explained by the small number of measures validated in this population and the lack of involvement of patients, their families and health and social care professionals in the development of existing measures.^86^ Patient involvement in PROM development and modification has been recommended by researchers and is a requirement in US Food and Drug Administration guidelines.^154^ Development or modification of PROMs should involve young adults throughout the research process to ensure PROMs are valid and accurately reflect the patient’s perspective.^155^ Existing measures do not comprehensively capture the symptoms and concerns affecting this population. Future research may take existing PROMs and modify them to improve the comprehensibility and relevance for young adults living with life-limiting conditions.

### Strengths and limitations of the study

A strength of this review is the broad and robust search strategy that was not limited by language or date restrictions. Whilst we used multiple databases to conduct our search we may not have captured the literature published within regional databases. We did not identify any papers published in a language other than English. This may have introduced geographic and linguistic bias and may affect the generalisability of the findings of this review.

We used a conceptual framework of palliative care to guide our synthesis of symptoms and concerns. In some instances, symptoms and concerns could have been placed into multiple categories (e.g. fatigue could be physical or psychological). Using a framework synthesis may have ‘detracted from the likely interconnectedness’ of symptoms and concerns across domains.^156^

We acknowledge that some of the PROMs identified aimed to measure a specific aspect of health (e.g. anxiety, depression) and did not aim to be comprehensive in their assessment of symptoms and concerns. But we did identify four PROMs with more than 80% content coverage of the identified symptom and concern themes in this review.

## Conclusion

Young adults living with life-limiting conditions experience a wide range of symptoms and concerns. We identified a large number of PROMs available for use in this population, but even the most comprehensive PROMs do not capture the holistic symptoms and concerns of young adults living with life-limiting conditions.

This review provides a conceptual framework of symptoms and concerns that can be used to develop or modify existing patient reported outcome measures for young adults living with life-limiting conditions.

## Supplemental Material

sj-docx-2-pmj-10.1177_02692163251405370 – Supplemental material for What are the symptoms and concerns of young adults living with life-limiting conditions and how well are they captured by patient reported outcome measures? A mixed-methods systematic review and framework synthesis Supplemental material, sj-docx-2-pmj-10.1177_02692163251405370 for What are the symptoms and concerns of young adults living with life-limiting conditions and how well are they captured by patient reported outcome measures? A mixed-methods systematic review and framework synthesis by Rachel L. Chambers, India Tunnard, Hannah Scott, Lorna K. Fraser and Katherine E. Sleeman in Palliative Medicine

sj-docx-3-pmj-10.1177_02692163251405370 – Supplemental material for What are the symptoms and concerns of young adults living with life-limiting conditions and how well are they captured by patient reported outcome measures? A mixed-methods systematic review and framework synthesis Supplemental material, sj-docx-3-pmj-10.1177_02692163251405370 for What are the symptoms and concerns of young adults living with life-limiting conditions and how well are they captured by patient reported outcome measures? A mixed-methods systematic review and framework synthesis by Rachel L. Chambers, India Tunnard, Hannah Scott, Lorna K. Fraser and Katherine E. Sleeman in Palliative Medicine

sj-docx-4-pmj-10.1177_02692163251405370 – Supplemental material for What are the symptoms and concerns of young adults living with life-limiting conditions and how well are they captured by patient reported outcome measures? A mixed-methods systematic review and framework synthesis Supplemental material, sj-docx-4-pmj-10.1177_02692163251405370 for What are the symptoms and concerns of young adults living with life-limiting conditions and how well are they captured by patient reported outcome measures? A mixed-methods systematic review and framework synthesis by Rachel L. Chambers, India Tunnard, Hannah Scott, Lorna K. Fraser and Katherine E. Sleeman in Palliative Medicine

sj-docx-5-pmj-10.1177_02692163251405370 – Supplemental material for What are the symptoms and concerns of young adults living with life-limiting conditions and how well are they captured by patient reported outcome measures? A mixed-methods systematic review and framework synthesis Supplemental material, sj-docx-5-pmj-10.1177_02692163251405370 for What are the symptoms and concerns of young adults living with life-limiting conditions and how well are they captured by patient reported outcome measures? A mixed-methods systematic review and framework synthesis by Rachel L. Chambers, India Tunnard, Hannah Scott, Lorna K. Fraser and Katherine E. Sleeman in Palliative Medicine

sj-pdf-1-pmj-10.1177_02692163251405370 – Supplemental material for What are the symptoms and concerns of young adults living with life-limiting conditions and how well are they captured by patient reported outcome measures? A mixed-methods systematic review and framework synthesis Supplemental material, sj-pdf-1-pmj-10.1177_02692163251405370 for What are the symptoms and concerns of young adults living with life-limiting conditions and how well are they captured by patient reported outcome measures? A mixed-methods systematic review and framework synthesis by Rachel L. Chambers, India Tunnard, Hannah Scott, Lorna K. Fraser and Katherine E. Sleeman in Palliative Medicine
