# Supplementary material for: What are the symptoms and concerns of young adults living with life-limiting conditions and how well are they captured by patient reported outcome measures? A mixed-methods systematic review and framework synthesis
Source: Palliat Med. 2026 Jan 13;40(3):314–32. doi: 10.1177/02692163251405370 (PMC12936152; doi:10.1177/02692163251405370)
Supplement: sj-docx-2-pmj-10.1177_02692163251405370 – Supplemental material for What are the symptoms and concerns of young adults living with life-limiting conditions and how well are they captured by patient reported outcome measures? A mixed-methods systematic review and framework synthesis [file sj-docx-2-pmj-10.1177_02692163251405370.docx]

*Supplementary file 2: Search strategy adapted from Knighting et al. (2021) [20] and Namisango et al. (2019) [14].*

| **Construct:** Life-limiting illness | \| 1. exp Palliative Care/ \| \| --- \| \| 1. exp Palliative Medicine/ \| \| 1. exp Terminally Ill/ \| \| 1. exp Heart Failure/ \| \| 1. exp MUSCULAR DYSTROPHY, DUCHENNE/ \| \| 1. exp Neoplasms/ \| \| 1. exp Muscular Dystrophies/ \| \| 1. exp Cerebral Palsy/ \| \| 1. exp Spinal Dysraphism/ \| \| 1. exp Cystic Fibrosis/ \| \| 1. exp Neurodegenerative Diseases/ \| \|  \| \| 1. exp Genetic Diseases, Inborn/ \| \| 1. exp Chromosome Disorders/ \| \| 1. (degenerative adj3 disease*).ti,ab. \| \| 1. (degenerative adj3 illness*).ti,ab. \| \| 1. (degenerative adj3 condition*).ti,ab. \| \| 1. (degenerative adj3 disorder*).ti,ab. \| \| 1. (degenerative adj3 abnormalit*).ti,ab. \| \| 1. (degenerative adj3 impairment*).ti,ab. \| \| 1. (progressive adj3 disease*).ti,ab. \| \| 1. (progressive adj3 illness*).ti,ab. \| \| 1. (progressive adj3 condition*).ti,ab. \| \| 1. (progressive adj3 disorder*).ti,ab. \| \| 1. (progressive adj3 abnormalit*).ti,ab. \| \| 1. (progressive adj3 impairment*).ti,ab. \| \| 1. diminished life expectancy.ti,ab. \| \| 1. limited life expectancy.ti,ab. \| \| 1. cancer*.ti,ab. \| \| 1. duchenne.ti,ab. \| \| 1. dying.ti,ab. \| \| 1. end of life.ti,ab. \| \| 1. end stage renal failure.mp. or end stage liver failure.ti,ab. [mp=title, book title, abstract, original title, name of substance word, subject heading word, floating sub-heading word, keyword heading word, organism supplementary concept word, protocol supplementary concept word, rare disease supplementary concept word, unique identifier, synonyms] \| \| 1. heart failure.ti,ab. \| \| 1. incurable.ti,ab. \| \| 1. Life-limit*.ti,ab. \| \| 1. Life limit*.ti,ab. \| \| 1. (life adj3 short*).ti,ab. \| \| 1. (live* adj3 short*).ti,ab. \| \| 1. life threaten*.ti,ab. \| \| 1. Limited life expectancy.ti,ab. \| \| 1. LLC.ti,ab. \| \| 1. LLI.ti,ab. \| \| 1. muscular dystroph*.ti,ab. \| \| 1. neoplasm*.ti,ab. \| \| 1. neurodegenerative condition*.ti,ab. \| \| 1. neurodegenerative disease*.ti,ab. \| \| 1. neurodegenerative illness*.ti,ab. \| \| 1. neurodegenerative disorder*.ti,ab. \| \| 1. neurodegenerative abnormalit*.ti,ab. \| \| 1. neurodegenerative impairment*.ti,ab. \| \| 1. oncology.ti,ab. \| \| 1. palliative.ti,ab. \| \| 1. poor prognosis.ti,ab. \| \| 1. (serious* adj3 ill*).ti,ab. \| \| 1. (terminal* adj3 ill*).ti,ab. \| \| 1. (terminal* adj3 care*).ti,ab. \| \| 1. (terminal* adj3 disease*).ti,ab. \| \| 1. (terminal* adj3 condition*).ti,ab. \| \| 1. (terminal* adj3 disorder*).ti,ab. \| \| 1. (terminal* adj3 abnormalit*).ti,ab. \| \| 1. (terminal* adj3 impairment*).ti,ab. \| \| 1. (genetic adj3 disease*).ti,ab. \| \| 1. (genetic adj3 disorder*).ti,ab. \| \| 1. (genetic adj3 illness*).ti,ab. \| \| 1. (genetic adj3 condition*).ti,ab. \| \| 1. (genetic adj3 abnormalit*).ti,ab. \| \| 1. (genetic adj3 impairment*).ti,ab. \| \| 1. (chromosomal adj3 disease*).ti,ab. \| \| 1. (chromosomal adj3 illness*).ti,ab. \| \| 1. (chromosomal adj3 disorder*).ti,ab. \| \| 1. (chromosomal adj3 condition*).ti,ab. \| \| 1. (Chromosomal adj3 abnormalit*).ti,ab. \| \| 1. (chromosomal adj3 impairment*).ti,ab. \| \| 1. (congenital adj3 disease*).ti,ab. \| \| 1. (congenital adj3 illness*).ti,ab. \| \| 1. (congenital adj3 disorder*).ti,ab. \| \| 1. (congenital adj3 condition*).ti,ab. \| \| 1. (congenital adj3 abnormalit*).ti,ab. \| \| 1. (congenital adj3 impairment*).ti,ab. \| \| 1. complex health* need*.ti,ab. \| \| 1. early death*.ti,ab. \| \| 1. cerebral pals*.ti,ab. \| \| 1. spina bifida.ti,ab. \| \| 1. cystic fibrosis.ti,ab. \| \| 1. encephalopath*.ti,ab. \| \| 1. impaired motor skill*.ti,ab. \| \| 1. spinal cord condition*.ti,ab. \| \|  \| \| 1. acquired brain injur*.ti,ab. \| \| 1. neurological condition*.ti,ab. \| \| 1. neuromuscular condition*.ti,ab. \| \| 1. multi-organ disease*.ti,ab. 2. 1 or 2 or 3 or 4 or 5 or 6 or 7 or 8 or 9 or 10 or 11 or 12 or 13 or 14 or 15 or 16 or 17 or 18 or 19 or 20 or 21 or 22 or 23 or 24 or 25 or 26 or 27 or 28 or 29 or 30 or 31 or 32 or 33 or 34 or 35 or 36 or 37 or 38 or 39 or 40 or 41 or 42 or 43 or 44 or 45 or 46 or 47 or 48 or 49 or 50 or 51 or 52 or 53 or 54 or 55 or 56 or 57 or 58 or 59 or 60 or 61 or 62 or 63 or 64 or 65 or 66 or 67 or 68 or 69 or 70 or 71 or 72 or 73 or 74 or 75 or 76 or 77 or 78 or 79 or 80 or 81 or 82 or 83 or 84 or 85 or 86 or 87 or 88 or 89 or 90 or 91 \| |
| --- | --- | --- | --- | --- | --- | --- | --- | --- | --- | --- | --- | --- | --- | --- | --- | --- | --- | --- | --- | --- | --- | --- | --- | --- | --- | --- | --- | --- | --- | --- | --- | --- | --- | --- | --- | --- | --- | --- | --- | --- | --- | --- | --- | --- | --- | --- | --- | --- | --- | --- | --- | --- | --- | --- | --- | --- | --- | --- | --- | --- | --- | --- | --- | --- | --- | --- | --- | --- | --- | --- | --- | --- | --- | --- | --- | --- | --- | --- | --- | --- | --- | --- | --- | --- | --- | --- | --- | --- | --- | --- | --- | --- | --- | --- |
| **Population:** Young adult | \| 1. young adult*.ti,ab. \| \| --- \| \| 1. young person.ti,ab. \| \| 1. young people.ti,ab. \| \| 1. youth*.ti,ab. \| \| 1. emerg* adult*.ti,ab. \| \| 1. early adult*.ti,ab. \| \| 1. exp Young Adult/ \| \| 1. exp ADOLESCENT/ 2. 93 OR 94 or 95 or 96 or 97 or 98 or 99 or 100 \| |
| **Outcome:**  Needs, symptoms and concerns | 1. Need*.ti 2. Concern*.ti 3. Symptom*.ti 4. 102 OR 103 OR 104 |
