# Supplementary material for: What are the symptoms and concerns of young adults living with life-limiting conditions and how well are they captured by patient reported outcome measures? A mixed-methods systematic review and framework synthesis
Source: Palliat Med. 2026 Jan 13;40(3):314–32. doi: 10.1177/02692163251405370 (PMC12936152; doi:10.1177/02692163251405370)
Supplement: sj-docx-3-pmj-10.1177_02692163251405370 – Supplemental material for What are the symptoms and concerns of young adults living with life-limiting conditions and how well are they captured by patient reported outcome measures? A mixed-methods systematic review and framework synthesis [file sj-docx-3-pmj-10.1177_02692163251405370.docx]

*Supplementary file 3: Characteristics of quantitative studies using patient reported outcome measures or questionnaires to capture pre-defined symptoms and concerns (n=66)*

| **Author, year** | **Country** | **Study design** | **Outcome measure** | **Aim(s)** | **Setting** | **Sample size** | **Age**  **(Mean, SD)** | **Gender** | **Race / Ethnicity** | **Hawker’s quality score [25]** |
| --- | --- | --- | --- | --- | --- | --- | --- | --- | --- | --- |
| Cancer | | | | | | | | | | |
| Abdelaal et al. 2021 [5] | Canada | Quantitative; retrospective chart review | Edmonton Symptom Assessment System Scale | To describe the demographics and symptom burden of AYA cancer patients who attended the integrated palliative care and psychiatry clinic, measure the impact of the clinic on AYAs’ symptom control, and examine their end-of-life outcomes | Hospital | 69 | 31.52 (6.3)  Range 17-39 | Female 59.4%  Male 40.6% | Not reported | 32; Good |
| Ameringer et al. 2022 [32] | United States | Quantitative; retrospective cohort study | Memorial Symptom Assessment Scale | 1) To examine the occurrence of symptoms in AYAs during active treatment  2) Describe symptoms in AYAs across cancer diagnostic groups by age group, sex, race/ethnicity, and time since diagnosis  3) Describe symptoms within diagnostic groups by age group, sex and time since diagnosis | Medical centres | 118 | 13-17 (47%)  18-29 (53%) | Female 42%  Male 58% | White 78%  Black/African-American 10%  Hispanic 7%  Other 5% | 33; Good |
| Ann-Yi et al. 2023 [33] | United States | Quantitative: retrospective review of clinical records | Edmonton Symptom Assessment Scale | To evaluate age variation in clinical and demographic factors of adult cancer patients receiving supportive care services | Hospital | 896 | 18-39 (33.1%) | Female 60%  Male 40% | White 49%  Hispanic 25%  Black 17%  Other/Unknown 9% | 30; Good |
| Bartolo et al. 2020 [34] | Portugal | Quantitative; cross-sectional | Reproductive Concerns After Cancer Scale  Fertility Problem Inventory  Hospital Anxiety and Depression Scale  The European Organisation for Research and Treatment of Cancer Quality-of-Life Questionnaire Core-30 | 1) To better understand the mechanisms involved in the maladjustment of young adult women faced with a reproduction-threatening disease  2) To explore the association between the representation of the importance of parenthood, fertility and parenthood concerns and depression, and health-related quality of life | Hospital | 104 | 36.61 (3.03) | Female 100% | Not reported | 30; Good |
| Chan et al. 2018 [35] | Singapore | Quantitative; longitudinal | Distress Thermometer  Rotterdam Symptom Checklist  Paediatric Quality of Life Generic Core Scales | 1) To evaluate Asian AYA patients' distress across a trajectory of three time points over a 6-month period following diagnosis  2) To evaluate the appropriateness of the distress thermometer to detect distress and symptom burden in Asian AYA cancer patients | Cancer centre | 65 | 27.8 (6.7%) | Female 44.6%  Male 55.4% | Chinese 75.4%  Malay 12.3%  Other 9.2%  Indian 3.1% | 34; Good |
| Dyson et al. 2012 [36] | Australia | Quantitative: cross-sectional survey | Beck Depression Inventory 2^nd^ edition – fast screen  State-Trait Anxiety Inventory  Supportive Care Needs Survey – Short Form | To explore psychological distress and unmet needs in adolescents and young adults (AYA) with cancer and identify predictors of distress among demographic and illness characteristics and supportive care needs | Cancer centre | 53 | 20.96 (SE 0.96)  Range 16-30 | Female 43%  Male 57% | Not reported | 31; Good |
| Graetz et al. 2019 [37] | United States | Quantitative: cross-sectional survey | Not applicable | To investigate AYAs' priorities during cancer, including psychosocial concerns, cure-directed therapy and potential late effects | Hospital | 203 | Range 15-29  15-17 (25%)  18-21 (20%)  22-29 (54%) | Female 46%  Male 54% | White 89%  Black / African American 3%  Asian / Pacific Islander 5%  Native American / Others 4% | 32; Good |
| Gupta et al. 2013 [38] | Canada | Quantitative: cross-sectional | Cancer Needs Questionnaire | To identify information and service needs important to YAs who are on, or soon after active therapy for cancer, and treated exclusively at an adult institution | Cancer centre | 243 | Median 28  Range 17-35 | Female 38.7%  Male 61.3% | Not reported | 29; Good |
| Gupta et al. 2023 [39] | Canada | Quantitative; retrospective cohort study | Edmonton Symptom Assessment System-revised | 1) To employ a multistate transition model to determine the prevalence, severity, and trajectory of symptoms in the cohort  2) To identify predictors of high symptom burden | Outpatient clinics | 4296 | Median 25  Range 15-29 | Female 48.6%  Male 51.4% | Not reported | 31; Good |
| Harper et al. 2023 [40] | United States | Quantitative; retrospective cohort | Edmonton Symptom Assessment System-revised | 1) To comprehensively describe symptom severity among adolescents and young adults with cancer at diagnosis and 1 year after diagnosis and identify demographic and clinical risk factors for higher severity  2) To evaluate symptoms trajectories among adolescents and young adults with cancer during the year following diagnosis  3) To compare symptom severity and trajectories with older adult patients with cancer" | Cancer centre | Diagnosis:  AYAs (18-39) 473  1 year after diagnosis:  AYAs 322 | Diagnosis:  32.12 (5.42)  1 year after diagnosis:  32.19 (5.57) | Diagnosis:  Female 52.2%  Male 47.8%  1 year after diagnosis:  Female 59.3%  Male 40.7% | Not reported | 29; Good |
| Hirayama et al. 2024 [38] | Japan | Quantitative; retrospective chart review | Distress thermometer and problem-list | To assess the feasibility of an ePRO tool for screening and determining the prevalence of distress and supportive care needs among AYAs | Hospital | 244 | 25.0 (7.6)  Range 15-39 | Female 50.8%  Male 49.2% | Not reported | 31; Good |
| Hughes et al. 2015 [39] | Australia | Quantitative; retrospective chart review | Edmonton Symptom Assessment Scale  Edmonton Classification System of Cancer Pain | To identify the prevalence, severity, and mechanism of pain and other symptoms in AYA patients referred to a palliative care service in a specialist Australian cancer centre | Cancer centre | 33 | 21.5 (2.3)  Range 16-24 | Female 64%  Male 36% | Not reported | 29; Good |
| Jonker-pool et al. 2004 [38] | Netherlands | Quantitative; cross-sectional survey | Not applicable | To assess the need for information or support concerning ‘sexual functioning after treatment’ of patients with testicular cancer, during treatment and at follow-up, by means of a questionnaire | Medical centres | Testicular cancer  264 | Testicular cancer  Median 36 | Male 100% | Not reported | 26; Fair |
| Kirchoff et al. 2017 [38] | United States | Quantitative: cross-sectional survey | Not applicable | To assess oncology providers’ perceptions of adolescent and young adult (AYA) cancer patients’ unmet need | Hospital | 91 healthcare professionals | 21.2 | Not reported | Not reported | 26; Fair |
| Lidington et al. 2021 [53] | United Kingdom | Quantitative; cross-sectional survey | Supportive care needs survey – long form  The European Organisation for Research and Treatment of Cancer Quality of Life Questionnaire Core 30  The Hospital Anxiety and Depression Scale  Illness Cognition Questionnaire | 1) To describe the unmet supportive care needs among YAs in each SCNS domain  2) To explore the relationship between supportive care need and clinical and demographic factors, HRQoL, psychological distress, illness cognitions, and service need using latent class analysis | Hospital | 317 | 36.2 (4.5) | Female 69.1%  Male 30.9% | White 85.8%  Asian/Asian British 8.2%  Mixed/multiple ethnic groups 3.8%  Other ethnic group 1.3%  Black/African/Caribbean/Black British 1.0% | 32; Good |
| Link et al. 2023 [43] | United States | Quantitative; retrospective cohort | Edmonton Symptom Assessment System-Revised  Canadian Problem Checklist | 1) To compare PROs data for AYAs and older adults (OAs) to better understand how the concerns of AYAs differ, which is key to providing individualized care and creating targeted programming and system-level change | Cancer centres | AYAs 2089 | 32.5 (5.28) | Female 57.3%  Male 42.7% | Not reported | 29; Good |
| Markwardt et al. 2024 [43] | United States | Quantitative; longitudinal | The Adolescent and Young Adult Psycho-Oncology Screening Tool (AYA-POST) | To compare distress and unmet needs between young adults (YAs) from three cancer clinics | Cancer clinics | 96 | 31.8 (5.64)  Range 19-39 | Female 64.6%  Male 33.3%  Prefer not to answer 2.1% | White 71.9%  Black 14.6%  More than one race 4.2%  Other 5.2%  Native Hawaiian / Pacific Islander 1.0%  Alaska Native 1.0%  Asian 1.0%  Prefer not to answer 1.0% | 33; Good |
| McCarthy et al. 2018 [45] | Australia | Quantitative; cross-sectional survey | Information needs  Posttraumatic stress disorder checklist  The Kessler psychological distress scale | 1) To explore health-related information needs of adolescent and young adults (AYAs) and their parent carers  2) To examine demographic and clinical variables associated with unmet information needs, including patient activation | Hospital | 196 | 21.6 (3.13)  Range 15-27 | Female 49% | Not reported | 31; Good |
| Millar et al. 2010 [46] | Australia | Quantitative; cross-sectional | Cancer needs questionnaire for young people  Depression, anxiety and stress scales | 1) To ascertain the most commonly-unmet needs of emerging adults with cancer, in various stages of time-since-treatment  2) To investigate links to psychological functioning | Hospital | 63 | 20.4  Range 18-24 | Female 63.5%  Male 36.5% | Not reported | 29; Good |
| Naik et al. 2020 [43] | Canada | Quantitative; retrospective cohort | PsychoSocial Scan for CANcer—Revised  (PSSCAN-R) questionnaire, which uses the Canadian Problem Checklist (CPC) | To evaluate the prevalence of emotional distress and psychosocial needs in young adult (aged 18–39) patients at the time of their breast cancer diagnosis compared to older patients | Cancer centre | 420 | Range 18-39 | Female 100% | Not reported | 26; Fair |
| Okamura et al. 2021 [47] | Japan | Quantitative; cross-sectional survey | The short-form Supportive Care Needs Survey questionnaire  The short-version multidimensional scale of perceived social support  The Kessler psychological distress scale  The EuroQoL 5 dimensions, 5 levels | 1) To describe unmet supportive care needs among young adults with cancer in Japan  2) To identify its associated factors | Outpatient clinic | 206 | 33.7 (4.3)  Range 22-39 | Female 87.4%  Male 12.6% | Not reported | 31; Good |
| Oosterhuis et al. 2008 [48] | United States | Quantitative; cross-sectional survey | Not applicable | To evaluate concerns about fertility-related side effects of cancer treatment in two samples: parents of pediatric cancer patients of all ages as well as adolescent cancer patients | Hospital | 37 | 18.3 (3.0) | Female 45.9%  Male 54.1% | Caucasian 59.5%  Latino/Hispanic 11%  Asian 11%  More than one ethnicity 11%  African American 5%  Other 2% | 31; Good |
| Ovayolu et al. 2013 [49] | Turkey | Quantitative; longitudinal | Edmonton Symptom Assessment Scale  Short Form - 36 | To assess the symptoms that may be encountered before and after a stem cell transplantation and quality of life | Hospital | 82 | 17-26 (31.7%)  27-36 (22.0%)  37-46 (15.9%)  47+ (30.5%) | Female 31.7%  Male 68.3% | Not reported | 28; Good |
| Pasek et al. 2016 [48] | Poland | Quantitative; cross-sectional survey | Berlin Social Support scale | To analyse the demand for support among cancer patients subjected to systemic treatment or radiotherapy | Cancer centre | 33 YAs | 19-39 (10% of sample) | Female 67.3%  Male 32.7% | Not reported | 22; Fair |
| Peersmann et al. 2022 [49] | Netherlands | Quantitative; cross-sectional survey | Insomnia Severity Index  Holland Sleep Disorder Questionnaire | 1) To estimate the prevalence of insomnia symptoms and daytime fatigue and to explore the co-occurrence of these symptoms in adolescents and young adults in follow-up after childhood cancer treatment  2) To assess risk factors of patients with (a) insomnia and daytime fatigue, (b) insomnia only, and (c) daytime fatigue only, compared to patients with no symptoms | Hospital | 576 | 17.0 (2.9)  18-26 (42.8%) | Female 49.6% | Not reported | 32; Good |
| Perl et al. 2016 [50] | Israel | Quantitative; cross-sectional survey | Short-Form 12  Cancer Rehabilitation Evaluation System  Sexual Functioning Summary Scale -Short Form  Cancer Survivors’ Unmet Needs Questionnaire | To characterize CRC patients’ specific needs and quality of life concerns | Medical centres | 50 | Median 35.5  Range 20-49 | Female 55%  Male 45% | Ashkenazi Jew 26%  Sepheradi Jew 38%  Mixed 10%  Former USSR 16%  Arab 4%  Other 2% | 33; Good |
| Pulewka et al. 2021 [51] | Germany | Quantitative; cross-sectional survey | Not applicable | To analyse demand for information and advice as well as medical, psychological, and social needs of adolescents and young adults (AYAs) and older patients (non-AYA) after hematopoietic stem cell transplantation (HSCT). | Hospital | 65 | Median 32 | Male YAs 56.7% | Not reported | 32; Good |
| Roberts et al. 1997 [50] | United States | Quantitative; cross-sectional | Brief Symptom Inventory | 1) To describe specific problems by young adult cancer patients with various diagnosis  2) To assess the life changes associated with the cancer and the relative intensity of these changes  3) To describe the patients' psychological wellbeing | Outpatient clinics | 46 | 31.4 (4.0)  Range 22-35 | Female 75% | White 92%  Hispanic 4%  African-American 2%  Asian 2% | 28; Good |
| Sanford et al. 2014 [52] | United States | Quantitative; prospective cohort | MD Anderson Symptom Inventory | To examine the symptom burden experienced by Yas receiving treatment for breast or colorectal cancer | Academic and community sites | 133 | 35 (4.3) | Female 83.8%  Male 14.3% | White 82.7%  Black 13.5% | 30; Good |
| Sawyer et al. 2017 [53] | Australia | Quantitative; cross-sectional survey | Cancer Needs Questionnaire for Parents and Carers of Young People with Cancer  The Posttraumatic Stress Disorder Checklist | 1) To determine how well cancer services are meeting some of the service needs of 15–25-year-old AYAs and their parent carers, both during cancer treatment and beyond.  2) To explore the association of unmet need with distress | Hospital | 196 | 21.6 (3.13) | Female 49% | Not reported | 31; Good |
| Sender et al. 2019 [54] | Germany | Quantitative; longitudinal | Supportive Care Needs Survey – Short Form  German version of the Distress Thermometer | To examine unmet supportive care needs and to investigate predictors of and changes in unmet needs over time | Hospital | 514 | 29.6 (6.1)  Range 18-39 | Female 75.1%  Male 24.9% | Not reported | 31; Good |
| Smith et al. 2013 [53] | United States | Quantitative; cross-sectional survey | Short Form health survey – 12  Paediatric Quality of Life Inventory | 1) To examine physical, emotional, social, cognitive, and psychological functioning of AYA patients / survivors with cancer  2) To determine potential disease, demographic, health care and symptom correlates of HRQOL | Cancer centre  Community hospital  Outpatient | 523 | 15-17 (4.4%)  18-24 (22.0%)  25-29 (24.1%)  30-24 (23.7%)  35-41 (25.8%) | Female 36.7%  Male 63.3% | White 59.3%  Hispanic 20.7%  Other 11.5%  Black 8.6% | 33; Good |
| Smrke et al. 2020 [54] | Canada | Quantitative; retrospective review of clinical records | Social network and support assessment tool  Canadian problem checklist | To investigate psychosocial needs of AYA compared to older adults with cancer at diagnosis | Cancer centre | 2045 AYAs | Median 33  Range 18-39 | Female 61.9%  Male 38.1% | Not reported | 31; Good |
| Soleimani et al. 2021 [55] | Canada | Quantitative; cross-sectional | Canadian problem checklist | 1) To evaluate whether AYA with germ cell tumour experience greater symptoms of clinical / subclinical anxiety than their non-AYA counterparts  2) To explore specific domains of patient reported psychosocial distress amongst AYA patients with a goal of identifying key resource development needs | Cancer centre | 349 | Median 33  Range 18-83 | Not reported | Not reported | 30; Good |
| Sun et al. 2019 [56] | China | Quantitative; cross-sectional | Fear of progression questionnaire – short form  General Anxiety Disorder questionnaire  Patient health questionnaire – 9 | To explore the prevalence and correlates of fear of cancer recurrence, anxiety and depressive symptoms in Chinese AYA cancer population | Hospital | 249 | 33.12 (4.82)  Range 17-39 | Female 68.3%  Male 31.7% | Not reported | 30; Good |
| Wettergren et al. 1999 [55] | Sweden | Quantitative; cross-sectional | The Impact of Event Scale  The Hospital Anxiety and Depression Scale  The Sense of Coherence Scale  The EORTC quality of life questionnaire | To evaluate the presence of post-traumatic stress symptoms (PTSS) in patients with haematological malignant disorders undergoing autologous stem cell transplantation (ASCT) | Hospital | 20 | 37  Range 17-54 | Female 40%  Male 60% | Not reported | 25; Fair |
| Zebrack 2008 [56] | United States  Canada | Quantitative; cross-sectional survey | Not applicable | To examine supportive care needs and preferences among young adult cancer patients | Not reported | 217 | 31.3 (5.4) | Female 85.7%  Male 12.9% | White/Caucasian 82.0%  Asian 4.6%  Hispanic/Latin 4.1%  Black/African American 3.7%  Native American 0.5% | 33; Good |
| Zebrack et al. 2013 [57] | United States | Quantitative; cross-sectional | Not applicable | 1) To assess AYA cancer patients’ reports of using information, emotional, and practical support services within the first 4 months of diagnosis, and the extent to which their desire for these services were not met.  2) To compare AYAs’ reports of services used and unmet needs across 3 age groups and across paediatric versus adult oncology settings | Hospital | 208 | 14-19 (46.6%)  20-29 (22.1%)  30-39 (31.3%) | Female 48.6%  Male 51.4% | White/Caucasian 43.3%  Hispanic/Latino 42.8%  Asian American 6.3%  African American 5.3%  American Indian 1.4% | 32; Good |
| Zebrack et al. 2014 [58] | United States | Quantitative; longitudinal | Brief Symptom Inventory – 18 | To identify trajectories of clinically significant levels of distress throughout the first year following diagnosis and to distinguish factors, including supportive care service use, that predict the extent to which AYAs report distress | Clinics | 215 | 23.6 (8.9) | Female 47.0%  Male 53.0% | Non-Hispanic White/Caucasian 44.2%  Hispanic/Latino 42.3%  Others 9.3%  Asian/Pacific Islander 6.0%  African American 5.1%  American Indian/Alaskan Native 1.4% | 33; Good |
| Cerebral Palsy | | | | | | | | | | |
| de Albuquerque Botura et al. 2017 [81] | Brazil | Quantitative: cross-sectional survey | Inventory of pain behaviours in neurological disability | To evaluate the presence of pain in patients diagnosed with severe cerebral palsy (CP) according to the degree of motor function impairment | Not reported | 93 | Median 23  Range 1-57 | Female 44.1%  Male 55.9% | Not reported | 30; Good |
| Congenital Heart Disease | | | | | | | | | | |
| Reid et al. 2008 [72] | Canada | Quantitative; retrospective chart review | Short Form - 36 | To examine the sexual behaviours and reproductive concerns among patients with moderate to complex congenital heart disease (CHD). | Cardiac centre | 321 | 16-17 (30%)  18-20 (70%) | Female 50%  Male 50% | Not reported | 26; Fair |
| Cystic Fibrosis | | | | | | | | | | |
| Dury et al. 2021 [62] | France | Quantitative: prospective cohort study | Unmet needs questionnaire | To identify the most frequent topics that CF adults need to discuss with health care teams during regular visits | Hospital | 50 | 27.6 (8.7) | Male 70% | Not reported | 31; Good |
| Friedman et al. 2018 [63] | United States | Quantitative; longitudinal | Patient Health Questionnaire-9  General Anxiety Disorder-7  The Memorial Symptom Assessment Scale – Cystic Fibrosis | To pilot a model of routine implementation of a CF-specific primary palliative care intervention, utilising a structured assessment of CF-associated symptoms, distress and coping to inform an individualised symptom management plan | Clinics | 41 | 31.9 (11.9)  Range 13-60 | Male 48.9% | Not reported | 30; Good |
| Goldbeck et al. 2010 [64] | Germany | Quantitative: case-control | Hospital Anxiety Depression Scale | To investigate the prevalence of symptoms of anxiety and depression in German-speaking patients with CF and the association of these symptoms to physical health status | Clinics | 670 | 23.1 (9.1)  Range 12-64 | Female 47.3%  Male 52.7% | Not reported | 31; Good |
| Hayee et al. 2019 [63] | United Kingdom | Quantitative; cross-sectional | Patient Health Questionnaire-9  Generalised Anxiety Disorder-7  Gastrointestinal Symptom Rating Scale  IBS Symptom Severity Score  Cystic Fibrosis Questionnaire-revised | To determine whether tools validated in GI disorders could describe the range and impact in cystic fibrosis | Hospital | 107 | 27.8 (9.6) | Female 56.1% | Not reported | 29; Good |
| Jaudszus et al. 2019 [64] | Germany | Quantitative; validation | Cystic Fibrosis Abdominal Score | 1) To confirm the conceptual framework and to assess the validity and reliability of the questionnaire  2) To assess whether items, domains, and general scores reflect what is important to patients (content validity) and whether differences exist between patients with CF and healthy controls (known-groups validity)  3) To explore the logical structure of the PROM | Hospital | 116 | Median 23.3 | Female 54.3%  Male 45.7% | Not reported | 29; Good |
| Knudson et al. 2016 [65] | Denmark | Quantitative: cross-sectional | The Major Depression Inventory  Cystic Fibrosis Questionnaire – Revised – Teen/Adult version | To examine the relationships among treatment adherence, symptoms of depression and health related quality of life in a population of young adults with cystic fibrosis | Hospital | 67 | 24.1  Range 18-30 | Female 59%  Male 40% | Not reported | 29; Good |
| Olveira et al. 2016 [66] | Spain | Quantitative; cross-sectional | Hospital Anxiety and Depression Scale Spanish version  Revised Cystic Fibrosis Questionnaire - Spain | 1) To assess self-reported symptoms of depression and anxiety in a cohort of patients with cystic fibrosis  2) To assess the relationship with health status and health-related quality of life | Cystic Fibrosis centres | 336 | 28.1 (8.2) | Female 48.2%  Male 51.8% | Not reported | 31; Good |
| Trandel et al. 2019 [67] | United States | Quantitative; cross-sectional | Edmonton Symptom Assessment Scale  Supportive Care Needs survey – short form 34 | 1) To determine the prevalence of existential distress in individuals with CF by identifying unmet existential needs  2) To investigate potential associations between symptom burden and unmet needs in this population | Cystic Fibrosis centres | 164 | Median 29  Range 18-66 | Female 44%  Male 56% | Caucasian 98%  Other or multiple races 2%  African-American 1% | 32; Good |
| Duchenne Muscular Dystrophy | | | | | | | | | | |
| Elsenbruch et al. 2013 [72] | Germany | Quantitative: cross-sectional survey | Depression Inventory for Children and Adolescents  Becks Depression Inventory  Health-related Quality of Life (DISABKIDS)  Short Form-36 | To address the impact of Duchenne muscular dystrophy (DMD) on self- reported health-related quality of life (HRQOL) and depressive symptoms in different age groups of patients to discern a possible need for improved psychosocial support or counselling | Hospital | 50 | 19.0 (0.5)  Range 8-23 | Not reported | Not reported | 29; Good |
| Houwen-vanOpstal et al. 2021 [73] | Netherlands | Quantitative; cross-sectional survey | The Utrecht Scale for Evaluation of Rehabilitation-Participation Restrictions scale | 1) To explore the prevalence of a broad variety of symptoms in the various stages of DMD (with and without steroid use)  2) To explore the prevalence of common secondary diagnoses  3) To evaluate the social participation level of patients with DMD older than 16 years of age  4) To explore correlations between social participation and symptoms | Neuromuscular centres | 84 | 22.0 (10.0)  Range 5-50 | Male 100% | Not reported | 31; Good |
| Facioscapulohumeral Muscular Dystrophy | | | | | | | | | | |
| Hamel et al. 2019 [62] | United States | Quantitative; cross-sectional survey | Not applicable | 1) To determine the frequency and relative importance of the most meaningful symptoms in facioscapulohumeral muscular dystrophy (FSHD)  2) To identify the demographic and clinical features that are associated with the greatest disease burden in this population" | Not reported | 328 | 54.5 (13.4)  Range 23-86 | Female 53.6%  Male 45.7%  Omitted 0.6% | White 95.7%  Asian 1.52%  Other 2.13%  Black 0.61% | 30; Good |
| HIV | | | | | | | | | | |
| Collins et al. 2007 [57] | Tanzania | Quantitative: prospective cohort study | Not applicable | To measure the prevalence of multidimensional palliative care needs of patients with HIV disease in Muheza, Tanzania | Hospital | 731 | 35.4 (13.7) | Female 70.9%  Male 30.6% | Not reported | 28; Good |
| Moens et al. 2015 [66] | South Africa  Uganda | Quantitative; cross-sectional | Memorial Symptom Assessment Scale – Short Form | 1) To identify clusters of subjects with similar combinations of symptoms  2) To describe the symptom combinations that distinguish the clusters  3) To compare the clusters with reference to treatment, demographic and symptom distress related characteristics | Home  Day care  Hospital  Inpatient | South Africa  70  Uganda  53 | South Africa  36.3 (8.8)  Uganda  36.8 (10.0) | South Africa  Female 77%  Uganda  60% | Not reported | 30; Good |
| Simms et al. 2013 [67] | Kenya  Uganda | Quantitative; survey | African Palliative Outcome Scale | To determine the prevalence, severity and risk factors for multidimensional problems in a population newly diagnosed with HIV at outpatient clinics in Africa | Outpatient clinics | 438 | 23.9  Range 18-59 | Female 61.6%  Male 38.4% | Not reported | 28; Good |
| Mixed population | | | | | | | | | | |
| Allen et al. 2022 [69]  NF1 36.9%  Cancer 22.6%  Sickle Cell Disease 22.5%  Primary immunodeficiency 16.7% | United States | Quantitative; cross-sectional survey | Not applicable | 1) To examine the needs and services of AYAs with chronic illnesses (i.e. cancer, SCD, NF1, and primary immunodeficiencies) and their caregivers  2) To explore the differences between AYAs with different diagnoses as well as among AYAs of different demographic groups, including age, sex and race  3) To highlight the key areas for improving transitional care and services and provide recommendations for those who treat AYAs with chronic illnesses | Hospital | 89 YAs | 23.49 (5.9)  Range 13-34 | Female 39.3%  Male 59.6%  Not disclosed 1.1% | White 41.6%  Black 30.3%  Asian 10.1%  Multiracial 9.0%  Not disclosed 5.6%  American Indian / Alaska Native 3.4% | 30; Good |
| Lewington et al. 2012 [79]  HIV/AIDS  Cancer  Heart failure  Renal failure  Liver failure  COPD | Uganda | Quantitative; cross-sectional and case review | African Palliative Outcome Scale | 1) To measure the magnitude of palliative care needs among hospital inpatients  2) To determine the point prevalence of inpatients with active life-limiting disease  3) To describe multidimensional need for palliative care among these patients | Hospital | Case review 122  PROMs  78 | 38 (15.89)  Range 13-80 | Female 64% | Not reported | 32; Good |
| Post-kidney transplant | | | | | | | | | | |
| Dahiya et al. 2020 [69] | India | Quantitative; cross-sectional survey | Depression, Anxiety and Stress Scale  World Health Organization Quality-of-Life Scale - BREF | To look at psychological symptoms, quality of life and adherence to immunosuppressive therapy in renal transplant recipients and their association with selected socio-demographic and clinical variables | Clinic | 96 | 38.7 (10.4) | Male 78.1% | Not reported | 29; Good |
| Sickle Cell Disease | | | | | | | | | | |
| Kelly et al. 2015 [72] | United States | Quantitative: cross-sectional | Profile of Mood States – Scale  Beck Depression Inventory | To evaluate internalizing symptoms in older adolescents with SCD and their demographically similar comparison peers without chronic illness | Home  Sickle cell centres | 48 | 18.2 (0.52) | Not reported | Black 95.8%  White 4.2% | 31; Good |
| Osunkwo et al. 2021 [73] | Canada; UK; France;  Netherlands;  Germany; USA; Italy;  Lebanon;  Bahrain;  Panama;  Brazil; Ghana;  Nigeria;  Oman; India  Saudi Arabia | Quantitative; cross-sectional survey | Not applicable | To provide real-world global insights into patients' views of the impact of SCD on their daily lives and the treatment they receive | Not reported | 2145 (1461 self-reported; 684 proxy-reported) | 24.7 (13.1)  Range 6-90 | Female 52% | Not reported | 28; Good |
| Robbins et al. 2020 [73] | United States | Quantitative; cross-sectional | Patient Health Questionnaire – 9  Generalised Anxiety Disorder – 7  Sickle Cell Self-Efficacy Scale  Brief Pain Inventory / PROMIS Pain Intensity Instrument | 1) To conduct systematic screening for depressive and anxiety symptoms, pain, and self-efficacy in an adult outpatient SCD clinic  2) To provide appropriate referral for psychological treatment among those identified to have mental health care needs  3) To document the prevalence of psychological symptoms in this population  4) To quantify success in linking patients to appropriate mental health care  5) To identify predictors of patients’ successful engagement in treatment | Outpatient medical centres | 336 | 33 (12.8) | Female 57.74% | African-American 98.51%  Other 1.49% | 30; Good |
| Sogutlu et al. 2011 [74] | United States | Quantitative; prospective cohort | Patient health questionnaire  Short form - 36 | To examine the impact of somatic symptom burden (SSB) on pain, depression, anxiety, health-care utilization, and quality-of-life in adults with sickle cell disease (SCD) | Academic and community sites | 230 | 34.4 (11.4)  Range 16-64 | Female 61.7%  Male 38.3% | Not reported | 29; Good |
| Spina Bifida | | | | | | | | | | |
| Bellin et al. 2013 [55] | United States | Quantitative; longitudinal | Hopkins Symptom Checklist-25 | To explore psychological symptoms in emerging adults with spina bifida and their association with self-management and satisfaction with family functioning | Clinics | Time 2: 48 | Time 2  22.04 (2.16)  Range 19-26 | Time 2  Female 54% | Time 2  Caucasian 77% | 32; Good |
| Bellin et al. 2010 [56] | United States | Quantitative; longitudinal | Hopkins Symptom Checklist-25 | To examine the unique relationships between multi-level ecological factors and psychological symptoms in young adults with spina bifida | Clinics | 61 | 21.05 (2.11)  Range 18-25 | Female 60.7% | Caucasian 77% | 32; Good |
| Choi et al. 2021 [57] | South Korea | Quantitative: cross-sectional survey | Not applicable | To identify the educational needs of AYAs with SB based on the discrepancies between perceived importance and proficiency levels during the transition process | Hospital | 108 | 19.88 (3.54)  Range 13-25 | Female 48.1%  Male 51.9% | Not reported | 30; Good |
| Dicianno et al. 2015 [58] | United States | Quantitative: retrospective chart review | Beck Depression Inventory-II | To examine the prevalence of depressive symptoms in adults with spina bifida and identify contributing factors for depressive symptomatology | Community | 190 | Mean 33.6 (11.1)  Range 18-77 | Female 54.2%  Male 45.8% | Caucasian 96.3%  African-American 2.1%  Unanswered 1.1%  Asian 0.5% | 32; Good |
