# Supplementary material for: What are the symptoms and concerns of young adults living with life-limiting conditions and how well are they captured by patient reported outcome measures? A mixed-methods systematic review and framework synthesis
Source: Palliat Med. 2026 Jan 13;40(3):314–32. doi: 10.1177/02692163251405370 (PMC12936152; doi:10.1177/02692163251405370)
Supplement: sj-docx-4-pmj-10.1177_02692163251405370 – Supplemental material for What are the symptoms and concerns of young adults living with life-limiting conditions and how well are they captured by patient reported outcome measures? A mixed-methods systematic review and framework synthesis [file sj-docx-4-pmj-10.1177_02692163251405370.docx]

| **Author, year** | **Diagnosis** | **Study design** | **Outcome measure** | **Physical** | **Psychological** | **Social** | **Spiritual** | **Other** |
| --- | --- | --- | --- | --- | --- | --- | --- | --- |
| Gwaltney et al. 2021 ^1^ | Barth syndrome | Qualitative; validation study, interviews | Barth Syndrome-Symptom Assessment | **Disease-related and treatment-related**  **symptoms**  Muscle weakness, lack of appetite, difficulty eating, vomiting, reflux, nausea, noise sensitivity, blurred vision, pain, fatigue, tiredness, physical developmental delay, low muscle tone, breathless, mouth sores, fever, dizziness, bone weakness, congestion, scoliosis, arthritis, dry eyes, hangnails, lack of energy, sleep  **Physical function**  Difficulties exercising | **Emotional function**  Sadness, depression  **Cognitive function**  Attention difficulty, memory problems, mental processing, confusion | **Interpersonal relationships**  Difficulty being socially active, going out with friends, dating  **Lifestyle restrictions**  Recreational activities. Independence  **Welfare**  Finances |  |  |
| Alander et al. 2021 ^2^ | Cancer | Qualitative; narrative analysis | Not applicable | **Disease-related and treatment-related symptoms**  Pain, incontinence, physical exhaustion. Effects of treatment on fertility, hair loss, changes to physical appearance  **Physical function**  Personal-care: showering and going to the bathroom | **Emotional function**  Grief due to infertility, stress, anxiety, worry, suicidal thoughts, fear of relapse  **Cognitive function**  Mental exhaustion  **Identity**  Illness identity | **Interpersonal relationships**  Social support, impact of fertility on their romantic relationship, isolation  **Disclosure**  Stigma | **Worry about death**  Thoughts about dying  **Existential concerns**  Hope/uncertainty of the future, acceptance that life is on hold | **Relationship with care providers and caring environment**  Interested, present and trustworthy, respected and included  **Managing health**  Correct, sufficient and clear information |
| Ameringer et al. 2015 ^3^ | Cancer | Mixed methods | Memorial Symptom Assessment Scale | **Disease-related and treatment-related symptoms**  Changes to taste, constipation, cough, diarrhoea, sleep, swallowing, dizziness, dry mouth, drowsiness, itching, lack of appetite, lack of energy, nausea, pain, difficulty urinating, breathlessness, skin changes, sweating, swelling, vomiting, weight loss, tingling in hands and feet. Hair loss | **Emotional function**  Irritable, nervous, sad, worrying  **Cognitive function**  Difficulty concentrating  **Identity**  Body-image |  |  |  |
| Avutu et al. 2022 ^4^ | Cancer | Qualitative; focus groups | Not applicable | **Disease-related and treatment-related symptoms**  Long-term treatment late effects, fertility, sexual health | **Identity**  Identity – forced transition to adulthood | **Interpersonal relationships**  Social isolation, peer support groups (with patients with similar conditions)  **Lifestyle restrictions**  Navigating social and professional life, loss of normalcy. Loss of independence | **Existential concerns**  Uncertainty about the future, life on hold/disrupted | **Relationship with care providers and caring environment**  Direct/honest communication, disclosure of sexuality, inclusivity, flexibility, acknowledging the role of partners and friends  **Managing health**  Involvement in decision making |
| Barakat et al. 2016 ^5^ | Cancer | Mixed methods | Not applicable | **Disease-related and treatment-related symptoms**  Nausea, fatigue. Hair loss | **Emotional function**  Stress, positive attitude, emotional about diagnosis  **Cognitive function**  Difficulty concentrating  **Identity**  Body image, confidence, feeling at a different point in life than peers/not having a ‘normal life’ | **Interpersonal relationships**  Peer support groups (with patients with similar conditions), supportive friends, supportive family, missing friends, supportive teachers, being a burden to others, romantic relationships  **Disclosure**  Explaining to others that they have cancer, being treated differently (baby-ed, bullied, extra attention), lack of understanding from friends and family  **Lifestyle restrictions**  Not being able to work or go to school, missing out (prom, camp, trip, college), lack of physical activity/giving up physical activities, giving up activities, losing autonomy/need for independence  **Welfare**  Financial difficulties | **Existential concerns**  Trying to plan for the future/feeling as if life is ‘on hold’ | **Managing health**  Written information about diagnosis, transitioning into adult services. |
| Choi et al. 2022 ^6^ | Cancer | Mixed methods (blogs) | Not applicable | **Disease-related and treatment-related symptoms**  Pain, fatigue, sleep, weight change, nausea, vomiting, hot flushes, sexual function, losing desire and libido, erectile dysfunction, vaginal dryness, hair loss, premature menopause, changes to physical appearance, fertility  **Physical function**  Physical activity, personal care (dressing), cooking, cleaning | **Emotional function**  Anxiety, fear, fear of recurrence, distress, guilt, depression, anger, grief, embarrassment or shame about side effects, post-traumatic stress, mood swings, suicidal  **Cognitive function**  Forgetting, word-finding, cognitive concerns, concentration, difficulty learning new things, taking longer to complete tasks  **Identity**  Illness identity, changes to body image, self-conscious, self-blame, self-identity, self-image, self-esteem, sexuality, loss of trust in their body | **Interpersonal relationships**  Being a burden to others, friends, loss of friendships, romantic relationships, isolated, parenting, peer support groups, friends disappearing  **Disclosure**  Lack of awareness in society, friends treating them differently due to diagnosis, scared and insecure to share diagnosis, treated differently due to diagnosis, disclosing infertility, hiding emotions from family to protect them, explaining cancer to children  **Lifestyle restrictions**  Uncertain about career, childcare, missing school/education  **Welfare**  Financial, insurance, housing | **Existential concerns**  Uncertainty about the future, existential despair, spiritual crisis | **Managing health**  Information on: fertility preservation, family planning, optimum nutrition, children’s health risk  **Relationship with care providers and caring environment**  Communication with healthcare professionals |
| David et al. 2012 ^7^ | Cancer | Qualitative: focus groups | Not applicable | **Disease-related and treatment-related symptoms**  Changes to physical appearance, side effects | **Emotional function**  Fear, upset, denial, optimism  **Identity**  Being different to other people with cancer | **Interpersonal relationships**  Treated the same, sense of belonging  **Lifestyle restrictions**  Independence  **Welfare**  Financial support services |  |  |
| Erickson et al. 2019 ^8^ | Cancer | Mixed methods | PROMIS Self-efficacy for managing symptoms scale | **Disease-related and treatment-related symptoms**  Lack of energy, nausea, drowsiness, sleep, pain. Hair loss |  |  |  |  |
| Hirayama et al. 2023 ^9^ | Cancer | Quantitative and qualitative; validation study | Japanese version of the distress thermometer and problem-list | **Disease-related and treatment-related symptoms**  Fatigue, pain, sleep, changes to physical appearance, difficulty eating, tingling in hands/feet, constipation, dry/itchy skin, dry/congested nose, nausea, diarrhoea, swelling, changes in urination, fever, indigestion, breathlessness, mouth sores, fertility, sexual issues  **Physical function**  Personal care (bathing, dressing) | **Emotional function**  Distressed, anxiety, worry, depression, nervous, fear, sadness, irritable  **Cognitive function**  Memory, concentration  **Identity**  Appearance/body image | **Interpersonal relationships**  Parents, romantic relationships, parenthood  **Lifestyle restrictions**  Childcare, work/school, loss of interest in usual activities  **Financial**  Financial, housing | **Connectedness**  Spiritual/religious concerns | **Managing health**  Information on diagnosis/treatment  **Relationship with care providers and caring environment**  Communication with healthcare professionals |
| Kohi et al. 2019 ^10^ | Cancer | Qualitative; focus groups | Not applicable | **Disease-related and treatment-related symptoms**  Pain, vomiting, loss of strength/energy. Fertility | **Emotional function**  Losing hope  **Identity**  Loss of identity | **Interpersonal relationships**  Community support  **Disclosure**  Stigma  **Lifestyle restrictions**  Reduced activities (sports, going to church, playing, work), education  **Welfare**  Financial impact of disease | **Worry about death**  Fearing death  **Connectedness**  Religion, faith |  |
| Lea et al. 2020 ^11^ | Cancer | Mixed methods | Not applicable | **Disease-related and treatment-related symptoms**  Fatigue, sexual health. Fertility  **Physical function**  Physical activity | **Emotional function**  Fear of recurrence, mental health  **Identity**  Body image, confidence | **Interpersonal relationships**  Peer support groups (with patients with similar conditions), interpersonal relationships  **Disclosure**  Labelled as someone with cancer  **Lifestyle restrictions**  Work, school  **Welfare**  Financial, insurance | **Connectedness**  Spiritual | **Managing health**  Information on: nutrition, diagnosis/treatment, late effects |
| Lidington et al. 2021 ^12^ | Cancer | Qualitative: interviews and focus groups | Not applicable | **Disease-related and treatment-related symptoms**  Fatigue, sexual function, hair loss  **Physical function**  Physical activity | **Emotional function**  Stressed, depression, wellbeing, fear of recurrence  **Identity**  Avoid cancer identity, confidence | **Interpersonal relationships**  Prioritising family, romantic relationships, isolation, distancing from friends.  **Disclosure**  Wanting to protect loved ones  **Lifestyle restrictions**  Work, childcare, loss of normalcy, balancing responsibilities, missing out (children/social life) | **Worry about death**  Facing mortality  **Existential concerns**  Uncertainty about the future, life is on hold  **Connectedness**  Religion | **Relationship with care providers and caring environment**  Feeling out of place in hospital (younger than other patients), hospital not designed for children to visit |
| Linder et al. 2019 ^13^ | Cancer | Mixed methods | Memorial Symptom Assessment Scale | **Disease-related and treatment-related symptoms**  Pain, lack of energy, sleep, nausea, drowsiness, changes to taste, lack of appetite, weight change, constipation, bloated, dry mouth, dizziness, hair loss, tingling in hands/feet  **Physical function**  Physical function | **Emotional function**  Irritable, worried, wellbeing, sad |  |  |  |
| Locatelli et al. 2023 ^14^ | Cancer | Quantitative; cross-sectional | Not applicable | **Disease-related and treatment-related symptoms**  Fatigue, lack of energy, nausea, pain, lack of appetite, sleep | **Emotional function**  Mood disturbance |  |  |  |
| Odh et al. 2016 ^15^ | Cancer | Qualitative; content analysis | Not applicable | **Disease-related and treatment-related symptoms**  Fatigue, nausea, lack of strength, lack of energy. Hair loss  **Physical function**  Stamina to look after children | **Emotional function**  Grief surrounding infertility, anxiety, fear, anger | **Interpersonal relationships**  Loss of social interactions, romantic relationships, loneliness  **Disclosure**  Lack of understanding from others  **Lifestyle restrictions**  Work/school, loss of normalcy | **Worry about death**  Death waiting for them  **Existential concerns**  Dreams ruined, losing their youth, uncertainty for the future  **Connectedness**  Religion, inner peace |  |
| Park et al. 2023 ^16^ | Cancer | Mixed methods | Not applicable | **Disease-related and treatment-related symptoms**  Nausea, vomiting, constipation. Fertility. Side-effects, changes to appearance, pain  **Physical function**  Exercise | **Emotional function**  Anxiety, depression, fear of death  **Identity**  Transition from healthy life to a cancer patient | **Interpersonal relationships**  Conflicts within families, social relationships (friends/coworkers), dating, sexual relationship, peer support groups (with patients with similar conditions)  **Lifestyle restrictions**  Work/school  **Welfare**  Financial support or advice | **Connectedness**  Religious conflicts  **Existential concerns**  Meaning/purpose of life, acceptance of disease | **Managing health**  Information on: disease, nutrition, fertility preservation, maintaining a healthy lifestyle, alternative therapy. Involvement in decision making. |
| Patterson et al. 2012 ^17^ | Cancer | Qualitative; focus groups and interviews | Not applicable | **Disease-related and treatment-related symptoms**  Pain, sleep. Fertility | **Emotional function**  Trauma, worry, unwanted thoughts, frightened, guilt for family distress  **Identity**  Confidence | **Interpersonal relationships**  Peer support groups (with patients with similar conditions), support from teachers/employers, friends, family, partners, fracturing romantic relationships, lonely.  **Lifestyle restrictions**  Work/school, disruptions to normal routines. Independence  **Welfare**  Managing finances, housing |  | **Relationship with care providers and caring environment**  Being treated like a child  Age-appropriate  **Managing health**  Information on alternative therapies/medicine |
| Rana et al. 2017 ^18^ | Cancer | Qualitative; focus groups and interviews | Not applicable | **Disease-related and treatment-related symptoms**  Fertility, effects on sex drive and hormones, hair loss | **Emotional function**  Distress, fear of recurrence  **Identity**  Self-esteem, sexuality | **Interpersonal relationships**  Peer support groups (with patients with similar conditions)  **Disclosure**  Sharing diagnosis with children | **Existential concerns**  Uncertainty about the future |  |
| Ruddy et al. 2013 ^19^ | Cancer | Qualitative; focus groups | Not applicable | **Disease-related and treatment-related symptoms**  Sexual dysfunction, lack of energy. Fertility, hair loss, weight gain, changes to physical appearance | **Cognitive function**  Cognitive difficulties | **Interpersonal relationships**  Peer support groups (with patients with similar conditions)  **Disclosure**  Communication difficulties with partner  **Lifestyle restrictions**  Education/career  **Welfare**  Financial |  |  |
| Ruddy et al. 2015 ^20^ | Cancer | Qualitative; interviews | Not applicable | **Disease-related and treatment-related symptoms**  Mouth sores, pain, weight changes, vaginal dryness, sexual function. Side-effects, fertility, changes to physical appearance | **Emotional function**  Fear, depression  **Cognitive function**  Difficulty remembering, finding words, expressing themselves  **Identity**  Body image, not feeling like themselves | **Interpersonal relationships**  Family support, peer support groups (with patients with similar conditions), isolated  **Disclosure**  Discussing cancer with new partner  **Lifestyle restrictions**  Flexible work environments/reduced workload | **Connectedness**  Faith, spirituality |  |
| Simon et al. 2023 ^21^ | Cancer | Mixed methods | Not applicable | **Disease-related and treatment-related symptoms**  Pain, nausea, constipation, fatigue, muscle weakness. Hair loss | **Emotional function**  Anxiety  **Identity**  Self-conscious | **Interpersonal relationships**  Peer support groups (with patients with similar conditions), support from family and friends  **Lifestyle restrictions**  Social/recreational activities, missing out, work/school, childcare, loss of normalcy | **Existential concerns**  Changes to hopes/dreams | **Managing health**  Being involved in decisions |
| Takeuchi et al. 2019 ^22^ | Cancer | Qualitative; medical records content analysis | Not applicable | **Disease-related and treatment-related symptoms**  Fertility | **Emotional function**  Distress, fear, worried  **Identity**  Afraid of using masculine/feminine role in relationship |  |  |  |
| Tan et al. 2024 ^23^ | Cancer | Mixed methods | Reproductive Concerns After Cancer scale | **Disease-related and treatment-related symptoms**  Fertility |  | **Disclosure**  Disclosing infertility with partner | **Existential concerns**  Adjusting expectations for the future (accepting a childless future) |  |
| Kubiak et al. 2023 ^24^ | Chronic kidney disease | Qualitative; interviews | Not applicable |  | **Emotional function**  Fear of recurrence  **Identity**  Body image, confidence | **Disclosure**  Bullying  **Lifestyle restrictions**  Work/school, support from parents |  | **Managing health**  Information on: family planning, impact of pregnancy on health |
| Chen et al. 2017 ^25^ | Congenital heart disease | Questionnaire; Delphi survey | Not applicable | **Disease-related and treatment-related symptoms**  Weight change  **Physical function**  Physical activity | **Emotional function**  Positive attitude towards illness  **Identity**  Body image, confidence  **Behaviour**  Substance use | **Interpersonal relationships**  Understanding from others, family support, support from friends, peer support groups  **Disclosure**  Communication with family and friends about illness, prejudice from others, public understanding of disease  **Lifestyle restrictions**  Work/school  **Welfare**  Financial | **Worry about death and dying**  Fear of death | **Relationship with care providers and caring environment**  Privacy, communication with healthcare professionals  **Managing health**  Information about the illness and course of the disease progression, prevention and management of symptoms, sharing information with patients/families/educators/employers, health self-management, transition to adult services, genetic counselling |
| Brissette et al. 1988 ^26^ | Cystic Fibrosis | Mixed methods | Not reported |  | **Emotional function**  Worried, burden of treatment | **Lifestyle restrictions**  Independence, autonomy, physical activity/recreation, school/work  **Disclosure**  Stigma  **Welfare**  Financial resources | **Existential concerns**  Setting appropriate goals | **Managing health**  Information on: birth control methods, impact of pregnancy on health, sterility and associated impotence, medication, causes of symptoms, interpretation of test results |
| Dellon et al. 2010 ^27^ | Cystic fibrosis | Qualitative; interviews | Not applicable | **Disease-related and treatment-related symptoms**  Shortness of breath, fatigue, pain, nausea, cough | **Emotional function**  Anxiety  **Behavioural**  Anorexia |  |  |  |
| Hailey et al. 2019 ^28^ | Cystic fibrosis | Qualitative; interviews | Cystic Fibrosis Questionnaire-Revised | **Disease-related and treatment-related symptoms**  Reproductive health, lack of energy | **Emotional function**  Stressed | **Interpersonal relationships**  Balancing role as a parent and patient, being a burden to others.  **Disclosure**  Parent-child communication (challenging questions related to death), impact of disclosure on children  **Lifestyle restrictions**  Childcare  **Welfare**  Finances |  | **Managing health**  Information on: impact of pregnancy on health, fertility, birth control, heritability of the disease. |
| Rosero et al. 2024 ^29^ | Duchenne muscular dystrophy | Mixed methods | Not applicable | **Disease-related and treatment-related symptoms**  Muscle weakness, stiffness, constipation, cough, diarrhoea, sleep, fatigue, pain, swallowing, headache, lack of energy, difficulty urinating, breathing difficulties, muscle cramps, gastrointestinal problems, swelling, tiredness  **Physical function**  Mobility | **Emotional function**  Mood, sad, anxiety, fear of disease worsening, anger  **Cognitive function**  Difficulty thinking and communicating, concentration  **Identity**  Body-image, confidence | **Interpersonal relationships**  Burden to others, childcare/parenting, friends  **Disclosure**  Treated differently  **Lifestyle restrictions**  Unable to do usual activities, missing out, physical activities, loss of interest in usual activities, independence |  | **Managing health**  Information |
| Jameson et al. 2008 ^30^ | HIV | Qualitative; interviews | Not applicable | **Disease-related and treatment-related symptoms**  Weight loss, pain, cough, weakness, fatigue, fever, vomiting, itchy/skin problems, nausea, diarrhoea, shortness of breath, changes to vision, sleep, dizziness | **Emotional function**  Sad, anxiety  **Cognitive function**  Confusion | **Interpersonal relationships**  Support from friends.  **Disclosure**  Lack of support from friends due to stigma  **Lifestyle restrictions**  Childcare  **Welfare**  Financial support | **Connectedness**  Religion, support from church members  **Existential concerns**  Uncertainty about the future |  |
| Selman et al. 2013 ^31^ | HIV | Qualitative; interviews | Not applicable | **Disease-related and treatment-related symptoms**  Pain, sleep. Side-effects, fertility  **Physical function**  Mobility | **Emotional function**  Distress, fear, worried | **Interpersonal relationships**  Isolated  **Disclosure**  Stigma  **Lifestyle restrictions**  Work/school  **Finances**  Not having enough food to eat | **Connectedness**  Religion, spirituality |  |
| Uwimana et al. 2007 ^32^ | HIV/AIDS | Mixed methods | Not applicable | **Disease-related and treatment-related symptoms**  Pain. Side-effects of medication |  | **Disclosure**  Stigma  **Lifestyle restrictions**  Work  **Welfare**  Financial assistance, housing | **Connectedness**  Spiritual support | **Managing health**  Information on nutrition, diagnosis. |
| Matthie et al. 2016 ^33^ | Sickle cell disease | Qualitative; interviews | Not applicable | **Disease-related and treatment-related symptoms**  Pain, fatigue, fever | **Emotional function**  Mental health, stress  **Identity**  Sexuality | **Interpersonal relationships**  Family, social roles, sexuality.  **Disclosure**  Fear of being judged, interruptions to family  **Lifestyle restrictions**  Loss of normalcy, education, employment  **Welfare**  Financial support | **Connectedness**  Religion | **Relationship with care providers and caring environment**  Stigma |
| Starowicz et al. 2021 ^34^ | Spina bifida | Quantitative; retrospective cohort chart review | Not applicable | **Disease-related and treatment-related symptoms**  Swallowing, swelling, sleep, cramping, seizure, bowel/bladder problems, bone health, menstruation, sexual function, headache, respiratory, incontinence, constipation, numbness, vision, speech clarity, seizures, pain, sexual health, weight changes, skin changes. Fertility  **Physical function**  Mobility, fitness, access to assistive devices (e.g. wheelchair) | **Emotional function**  Affective disorders  **Behavioural**  Substance use  **Cognitive function**  Speech | **Lifestyle restrictions**  Peer support groups (with patients with similar conditions)  , work/school, leisure activities, physical activities/fitness, social support, independence  **Welfare**  Financial |  |  |

1. Gwaltney C, Stokes J, Aiudi A, et al. Development and content validity of the Barth Syndrome Symptom Assessment (BTHS-SA) for adolescents and adults. Orphanet Journal of Rare Diseases 2021; 16(1); 264.

2. Alander MEJ, Klaeson K, Nyqvist et al. Lived experiences and caring needs in young adults diagnosed with cancer. In Nursing Forum 2021 (Vol 56, No 4, 781-790).

3. Ameringer S, Elswick Jr RK, Stegenda K, et al. Symptom profiles of adolescents and young adults in active cancer treatment by diagnostic groups. Cancer nursing 2022; 45(4); 306-315.

4. Avutu V, Lynch KA, Barnett ME, et al. Psychosocial needs and preferences for care among adolescent and young adult cancer patients (ages 15–39): A qualitative study. Cancers 2022, 14(3), 710.

5. Barakat LP, Galtieri LR, Szalda D, et al. Assessing the psychosocial needs and program preferences of adolescents and young adults with cancer. Supportive Care in Cancer 2016, 24, 823-832.

6. Choi E, Becker H, Kim S. Unmet needs in adolescents and young adults with cancer: A mixed-method study using social media. Journal of Pediatric Nursing 2022; 64; 31-41.

7. David CL, Williamson K, Tilsley DO. A small scale, qualitative focus group to investigate the psychosocial support needs of teenage young adult cancer patients undergoing radiotherapy in Wales. European Journal of Oncology Nursing 2012; 16(4); 375-379.

8. Erickson JM, Ameringer S, Linder L, et al. Using a heuristic app to improve symptom self-management in adolescents and young adults with cancer. Journal of Adolescent and Young Adult Oncology 2019; 8(2); 131-141.

9. Hirayama T, Fujimori M, Yanai Y, et al. Development and evaluation of the feasibility, validity, and reliability of a screening tool for determining distress and supportive care needs of adolescents and young adults with cancer in Japan. Palliative & Supportive Care 2023; 21(4); 677-687.

10. Kohi TW, von Essen L, Masika GM, et al. Cancer-related concerns and needs among young adults and children on cancer treatment in Tanzania: a qualitative study. BMC cancer 2019; 19; 1-9.

11. Lea S, Martins A, Fern LA, et al. The support and information needs of adolescents and young adults with cancer when active treatment ends. BMC cancer 2020; 20; 1-13.

12. Lidington E, Vlooswijk C, Stallard K, et al. ‘This is not part of my life plan’: A qualitative study on the psychosocial experiences and practical challenges in young adults with cancer age 25 to 39 years at diagnosis. European Journal of Cancer Care 2021; 30(5).

13. Linder LA, Stegenga K, Erickson J, et al. Priority symptoms, causes, and self-management strategies reported by AYAs with cancer. Journal of pain and symptom management 2019; 58(5); 774-783.

14. Locatelli G, Pasta A, Bentsen L, et al. Symptom Patterns in Young Adults with Cancer: An App-Based Study. In Seminars in Oncology Nursing 2023; 39(5).

15. Odh I, Löfving M, Klaeson K. Existential challenges in young people living with a cancer diagnosis. European Journal of Oncology Nursing 2016; 24; 54-60.

16. Park M, Kwon SY, Yun H, et al. Care needs of adolescents and young adults with cancer undergoing active treatment in South Korea: a mixed methods study. Journal of Adolescent and Young Adult Oncology 2023; 12(3); 398-407.

17. Patterson P, Millar B, Desille N, et al. The unmet needs of emerging adults with a cancer diagnosis: a qualitative study. Cancer Nursing 2012; 35(3); E32-E40.

18. Rana P, Ratcliffe J, Sussman J, et al. Young women with breast cancer: needs and experiences. Cogent Medicine 2017; 4(1).

19. Ruddy KJ, Greaney ML, Sprunck-Harrild K, et al. Young women with breast cancer: a focus group study of unmet needs. Journal of adolescent and young adult oncology 2013; 2(4); 153-160.

20. Ruddy KJ, Greaney ML, Sprunck-Harrild K, et al. A qualitative exploration of supports and unmet needs of diverse young women with breast cancer. J Community Support Oncology 2015; 13(9); 323-9.

21. Simon PJ, Pyke-Grimm KA, Nasr AS. Assessing the Needs of Adolescents and Young Adults Receiving Cancer Treatment: A Mixed Methods Study. Journal of Adolescent and Young Adult Oncology 2023; 12(1); 43-52.

22. Takeuchi E, Shimizu M, Miyata K, et al. A content analysis of multidimensional support needs regarding fertility among cancer patients: How can nonphysician health care providers support?. Journal of adolescent and young adult oncology 2019; 8(2); 205-211.

23. Tan CY, Francis-Levin N, Stelmak D, et al. Differentiating gender-based reproductive concerns among adolescent and young adult cancer patients: a mixed methods study. Journal of Psychosocial Oncology 2024; 42(4); 526-542.

24. Kubiak N, Fehrenbach C, Prüfe J, et al. Do we need palliative care in pediatric nephrology? Patients’ and caregivers’ disease experience. Children 2023; 10(2); 324.

25. Chen CW, Su WJ, Chiang YT, et al. Healthcare needs of adolescents with congenital heart disease transitioning into adulthood: a Delphi survey of patients, parents, and healthcare providers. European Journal of Cardiovascular Nursing 2017; 16(2); 125-135.

26. Brissette S, Zinman R, Reidy M. Disclosure of psychosocial concerns of young adults with advanced cystic fibrosis (CF) by a nurse home visiting program. International Journal of Nursing Studies 1988; 25(1); 67-72.

27. Dellon EP, Shores MD, Nelson KI, et al. Family caregiver perspectives on symptoms and treatments for patients dying from complications of cystic fibrosis. Journal of pain and symptom management 2010; 40(6); 829-837.

28. Hailey CE, Tan JW, Dellon EP, et al. Pursuing parenthood with cystic fibrosis: reproductive health and parenting concerns in individuals with cystic fibrosis. Pediatric pulmonology 2019; 54(8); 1225-1233.

29. Rosero S, Weinstein J, Seabury J, et al. Patient‐and caregiver‐reported impact of symptoms in Duchenne muscular dystrophy. Muscle & Nerve 2024.

30. Jameson C. The palliative care needs of patients with stage 3 and 4 HIV infection. Indian Journal of Palliative Care 2008; 14(1); 1.

31. Selman L, Simms V, Penfold S, et al. 'My dreams are shuttered down and it hurts lots’–a qualitative study of palliative care needs and their management by HIV outpatient services in Kenya and Uganda. BMC Palliative Care 2013; 12; 1-13.

32. Uwimana J, Struthers P. Met and unmet palliative care needs of people living with HIV/AIDS in Rwanda. SAHARA-J: Journal of Social Aspects of HIV/AIDS 2007; 4(1); 575-585.

33. Matthie N, Hamilton J, Wells D, et al. Perceptions of young adults with sickle cell disease concerning their disease experience. Journal of Advanced Nursing 2016; 72(6); 1441-1451.

34. Starowicz J, Cassidy C, Brunton L. Health concerns of adolescents and adults with spina bifida. Frontiers in neurology 2021; 12.
