# Supplementary material for: What are the symptoms and concerns of young adults living with life-limiting conditions and how well are they captured by patient reported outcome measures? A mixed-methods systematic review and framework synthesis
Source: Palliat Med. 2026 Jan 13;40(3):314–32. doi: 10.1177/02692163251405370 (PMC12936152; doi:10.1177/02692163251405370)
Supplement: sj-docx-5-pmj-10.1177_02692163251405370 – Supplemental material for What are the symptoms and concerns of young adults living with life-limiting conditions and how well are they captured by patient reported outcome measures? A mixed-methods systematic review and framework synthesis [file sj-docx-5-pmj-10.1177_02692163251405370.docx]

Supplementary file 5. Comprehensiveness of each patient reported outcome measure in capturing the symptoms and concerns important to young adults living with life-limiting conditions

| Patient reported outcome measure | **Physical** | | | **Psychological** | | | **Social** | | | | **Spiritual** | | | **Quality healthcare** | | Content coverage |
| --- | --- | --- | --- | --- | --- | --- | --- | --- | --- | --- | --- | --- | --- | --- | --- | --- |
|  | Disease-related symptoms | Treatment-related symptoms | Physical function | Emotional function | Cognitive function | Identity | Interpersonal relationships | Disclosure | Lifestyle restrictions | Welfare | Worry about death and dying | Connectedness to religion, faith and spirituality | Existential concerns | Relationship with care providers and caring environment | Managing health: Involvement in decision making and information |  |
| Cancer Rehabilitation Evaluation System |  |  |  |  |  |  |  |  |  |  |  |  |  |  |  | 93.3%; High/strong |
| Cancer Survivors Unmet Needs Questionnaire |  |  |  |  |  |  |  |  |  |  |  |  |  |  |  | 80.0%; High/strong |
| Supportive Care Needs Survey – Long Form |  |  |  |  |  |  |  |  |  |  |  |  |  |  |  | 80.0%; High/strong |
| The Cancer Assessment for Young Adults – Testicular (CAYA-T) |  |  |  |  |  |  |  |  |  |  |  |  |  |  |  | 80.0%; High/strong |
| World Health Organisation Quality of Life Scale - BREF |  |  |  |  |  |  |  |  |  |  |  |  |  |  |  | 73.3%; Moderate |
| Quality of Life – Cancer Survivors (QOL-CS) |  |  |  |  |  |  |  |  |  |  |  |  |  |  |  | 66.7%; Moderate |
| Canadian Problem Checklist |  |  |  |  |  |  |  |  |  |  |  |  |  |  |  | 60.0%; Moderate |
| Cancer Distress Scales for Adolescents and Young Adults (CDS-AYA) |  |  |  |  |  |  |  |  |  |  |  |  |  |  |  | 60.0%; Moderate |
| Cancer Needs Questionnaire – Young People (CNQ-YP) |  |  |  |  |  |  |  |  |  |  |  |  |  |  |  | 60.0%; Moderate |
| Health related quality of life - DISABKIDS |  |  |  |  |  |  |  |  |  |  |  |  |  |  |  | 60.0%; Moderate |
| The Ferrans and Powers Quality of Life Index (QLI): Cancer III Versions |  |  |  |  |  |  |  |  |  |  |  |  |  |  |  | 60.0%; Moderate |
| Supportive Care Needs Survey – Short Form |  |  |  |  |  |  |  |  |  |  |  |  |  |  |  | 60.0%; Moderate |
| African Palliative Care Association Palliative Outcome Scale |  |  |  |  |  |  |  |  |  |  |  |  |  |  |  | 53.3%; Moderate |
| AYA Needs Assessment and Service Bridge |  |  |  |  |  |  |  |  |  |  |  |  |  |  |  | 53.3%; Moderate |
| Beck’s Depression Inventory |  |  |  |  |  |  |  |  |  |  |  |  |  |  |  | 53.3%; Moderate |
| Distress Thermometer and Problem List |  |  |  |  |  |  |  |  |  |  |  |  |  |  |  | 53.3%; Moderate |
| Cystic Fibrosis Questionnaire |  |  |  |  |  |  |  |  |  |  |  |  |  |  |  | 53.3%; Moderate |
| Cystic Fibrosis Questionnaire – Teen/Adult Version |  |  |  |  |  |  |  |  |  |  |  |  |  |  |  | 53.3%; Moderate |
| EORTC-QLQ-C30 |  |  |  |  |  |  |  |  |  |  |  |  |  |  |  | 53.3%; Moderate |
| Life Impact Scale |  |  |  |  |  |  |  |  |  |  |  |  |  |  |  | 53.3%; Moderate |
| Young Adult Psychosocial Assessment Strategy (YA-PAS) |  |  |  |  |  |  |  |  |  |  |  |  |  |  |  | 53.3% Moderate |
| Adult Behaviour Checklist (ABCL) |  |  |  |  |  |  |  |  |  |  |  |  |  |  |  | 46.7%; Moderate |
| Brief Pain Inventory |  |  |  |  |  |  |  |  |  |  |  |  |  |  |  | 46.7%; Moderate |
| Brief Symptom Inventory |  |  |  |  |  |  |  |  |  |  |  |  |  |  |  | 46.7%; Moderate |
| Fear of Progression Questionnaire – Short Form |  |  |  |  |  |  |  |  |  |  |  |  |  |  |  | 46.7%; Moderate |
| Functional Assessment of Cancer Therapy – General Scale (FACT-G) |  |  |  |  |  |  |  |  |  |  |  |  |  |  |  | 46.7%; Moderate |
| Health Promoting Lifestyle Profile II (HPLP-II) |  |  |  |  |  |  |  |  |  |  |  |  |  |  |  | 46.7%; Moderate |
| MD Anderson Symptom Inventory |  |  |  |  |  |  |  |  |  |  |  |  |  |  |  | 46.7%; Moderate |
| Paediatric Quality of Life Inventory (PedsQL) 4.0 |  |  |  |  |  |  |  |  |  |  |  |  |  |  |  | 46.7%; Moderate |
| Paediatric Quality of Life Inventory (PedsQL) 4.0 Generic Core Scales |  |  |  |  |  |  |  |  |  |  |  |  |  |  |  | 46.7%; Moderate |
| Perceived Barriers Scale |  |  |  |  |  |  |  |  |  |  |  |  |  |  |  | 46.7%; Moderate |
| PROMIS Self-Efficacy for Managing Symptoms Scale (PROMIS S-E) |  |  |  |  |  |  |  |  |  |  |  |  |  |  |  | 46.7%; Moderate |
| Rotterdam Symptom Checklist |  |  |  |  |  |  |  |  |  |  |  |  |  |  |  | 46.7%; Moderate |
| Short Form-12 |  |  |  |  |  |  |  |  |  |  |  |  |  |  |  | 46.7%; Moderate |
| Short Form-36 |  |  |  |  |  |  |  |  |  |  |  |  |  |  |  | 46.7%; Moderate |
| The Post-Traumatic Diagnostic Scale (PDS) |  |  |  |  |  |  |  |  |  |  |  |  |  |  |  | 46.7%; Moderate |
| Unmet Needs Questionnaire |  |  |  |  |  |  |  |  |  |  |  |  |  |  |  | 46.7%; Moderate |
| Young Adult Quality of Life Inventory (PedsQL) |  |  |  |  |  |  |  |  |  |  |  |  |  |  |  | 46.7%; Moderate |
| Depression Inventory for Children and Adolescents (DIKJ) |  |  |  |  |  |  |  |  |  |  |  |  |  |  |  | 40.0%; Moderate |
| Health Competence Beliefs Inventory (HCBI) |  |  |  |  |  |  |  |  |  |  |  |  |  |  |  | 40.0%; Moderate |
| Patient Health Questionnaire-9 |  |  |  |  |  |  |  |  |  |  |  |  |  |  |  | 40.0%; Moderate |
| Post-Traumatic Stress Disorder Checklist – Civilian Version (PCL-C) |  |  |  |  |  |  |  |  |  |  |  |  |  |  |  | 40.0%; Moderate |
| Posttraumatic Stress Disorder Checklist (PCL-S) |  |  |  |  |  |  |  |  |  |  |  |  |  |  |  | 40.0%; Moderate |
| Reproductive Concerns After Cancer Scale |  |  |  |  |  |  |  |  |  |  |  |  |  |  |  | 40.0%; Moderate |
| Spinal Muscular Atrophy – Health Index (SMA-HI) |  |  |  |  |  |  |  |  |  |  |  |  |  |  |  | 40.0%; Moderate |
| Symptom Checklist-90 Revised (SCL-90) |  |  |  |  |  |  |  |  |  |  |  |  |  |  |  | 40.0%; Moderate |
| UCLA Post Traumatic Stress Disorder Reaction Index |  |  |  |  |  |  |  |  |  |  |  |  |  |  |  | 40.0%; Moderate |
| Brief Fatigue Inventory (BFI) |  |  |  |  |  |  |  |  |  |  |  |  |  |  |  | 33.3%; Moderate |
| Brief Symptom Inventory - 18 (BSI-18) |  |  |  |  |  |  |  |  |  |  |  |  |  |  |  | 33.3%; Moderate |
| Center for Epidemiologic Studies Depression Scale (CES-D Scale) |  |  |  |  |  |  |  |  |  |  |  |  |  |  |  | 33.3% Moderate |
| EQ-5D |  |  |  |  |  |  |  |  |  |  |  |  |  |  |  | 33.3%; Moderate |
| Memorial Symptom Assessment Scale |  |  |  |  |  |  |  |  |  |  |  |  |  |  |  | 33.3%; Moderate |
| Memorial Symptom Assessment Scale – Short Form |  |  |  |  |  |  |  |  |  |  |  |  |  |  |  | 33.3%; Moderate |
| Major Depression Inventory |  |  |  |  |  |  |  |  |  |  |  |  |  |  |  | 33.3% Moderate |
| Multidimensional Fatigue Inventory (MFI-20) |  |  |  |  |  |  |  |  |  |  |  |  |  |  |  | 33.3%; Moderate |
| Sexual Functioning Summary Scale – Short Form |  |  |  |  |  |  |  |  |  |  |  |  |  |  |  | 33.3%; Moderate |
| Depression, Anxiety and Stress Scale |  |  |  |  |  |  |  |  |  |  |  |  |  |  |  | 26.7%; Low/weak |
| Edmonton Classification System of Cancer Pain |  |  |  |  |  |  |  |  |  |  |  |  |  |  |  | 26.7%; Low/weak |
| Fertility Problem Inventory |  |  |  |  |  |  |  |  |  |  |  |  |  |  |  | 26.7%; Low/weak |
| Insomnia Severity Index |  |  |  |  |  |  |  |  |  |  |  |  |  |  |  | 26.7%; Low/weak |
| Inventory of Pain Behaviours in Neurological Disorders |  |  |  |  |  |  |  |  |  |  |  |  |  |  |  | 26.7%; Low/weak |
| Memorial Symptom Assessment Scale – Cystic Fibrosis |  |  |  |  |  |  |  |  |  |  |  |  |  |  |  | 26.7%; Low/weak |
| Mishel Uncertainty in Illness Scale – Community (MUIS-C) |  |  |  |  |  |  |  |  |  |  |  |  |  |  |  | 26.7%; Low/weak |
| Multidimensional  Fatigue Symptom Inventory–Short Form (MFSI-SF) |  |  |  |  |  |  |  |  |  |  |  |  |  |  |  | 26.7%; Low/weak |
| Paediatric Quality of Life Inventory (PedsQL) 3.0 Cancer Module Adolescent Form |  |  |  |  |  |  |  |  |  |  |  |  |  |  |  | 26.7%; Low/weak |
| PROMIS Fatigue - Short Form 7a |  |  |  |  |  |  |  |  |  |  |  |  |  |  |  | 26.7%; Low/weak |
| The Post Traumatic Growth Inventory (PTGI) |  |  |  |  |  |  |  |  |  |  |  |  |  |  |  | 26.7%; Low/weak |
| Child Attitude Toward Illness Scale |  |  |  |  |  |  |  |  |  |  |  |  |  |  |  | 20.0%; Low/weak |
| Cystic Fibrosis Abdominal Pain |  |  |  |  |  |  |  |  |  |  |  |  |  |  |  | 20.0%; Low/weak |
| Edmonton Symptom Assessment Scale |  |  |  |  |  |  |  |  |  |  |  |  |  |  |  | 20.0%; Low/weak |
| Edmonton Symptom Assessment Scale – Revised (ESAS-r) |  |  |  |  |  |  |  |  |  |  |  |  |  |  |  | 20.0%; Low/weak |
| Fatigue Questionnaire (FQ) |  |  |  |  |  |  |  |  |  |  |  |  |  |  |  | 20.0%; Low/weak |
| Growth Through Uncertainty Scale (GTUS) |  |  |  |  |  |  |  |  |  |  |  |  |  |  |  | 20.0%; Low/weak |
| Illness Cognition Questionnaire |  |  |  |  |  |  |  |  |  |  |  |  |  |  |  | 20.0%; Low/weak |
| Impact of Events Scale |  |  |  |  |  |  |  |  |  |  |  |  |  |  |  | 20.0%; Low/weak |
| Pittsburgh Sleep Quality Index |  |  |  |  |  |  |  |  |  |  |  |  |  |  |  | 20.0%; Low/weak |
| Profile of Mood States |  |  |  |  |  |  |  |  |  |  |  |  |  |  |  | 20.0%; Low/weak |
| PROMIS Self-Efficacy for Managing Social Interactions—short form 8a (v1.0) |  |  |  |  |  |  |  |  |  |  |  |  |  |  |  | 20.0%; Low/weak |
| Sense of Coherence Scale |  |  |  |  |  |  |  |  |  |  |  |  |  |  |  | 20.0%; Low/weak |
| Sickle Cell Self-Efficacy Scale |  |  |  |  |  |  |  |  |  |  |  |  |  |  |  | 20.0%; Low/weak |
| The Bern Subjective Wellbeing Questionnaire for Adolescents |  |  |  |  |  |  |  |  |  |  |  |  |  |  |  | 20.0%; Low/weak |
| Beck Depression Inventory – Fast Screen |  |  |  |  |  |  |  |  |  |  |  |  |  |  |  | 13.3%; Low/weak |
| Behaviour Rating Inventory of Executive Function – Adult Version (BRIEF-A) |  |  |  |  |  |  |  |  |  |  |  |  |  |  |  | 13.3%; Low/weak |
| Fatigue Thermometer |  |  |  |  |  |  |  |  |  |  |  |  |  |  |  | 13.3%; Low/weak |
| Frankfurt Self-Concept Scale |  |  |  |  |  |  |  |  |  |  |  |  |  |  |  | 13.3%; Low/weak |
| Gastrointestinal Symptom Rating Scale |  |  |  |  |  |  |  |  |  |  |  |  |  |  |  | 13.3%; Low/weak |
| General Self-Efficacy Scale |  |  |  |  |  |  |  |  |  |  |  |  |  |  |  | 13.3%; Low/weak |
| Hospital Anxiety and Depression Scale (HADS) |  |  |  |  |  |  |  |  |  |  |  |  |  |  |  | 13.3%; Low/weak |
| Holland Sleep Disorder Questionnaire |  |  |  |  |  |  |  |  |  |  |  |  |  |  |  | 13.3%; Low/weak |
| IBS Symptom Severity Scale |  |  |  |  |  |  |  |  |  |  |  |  |  |  |  | 13.3%; Low/weak |
| JenAbdomen-CF Score |  |  |  |  |  |  |  |  |  |  |  |  |  |  |  | 13.3%; Low/weak |
| Metacognitions Questionnaire – 30 (MCQ-30) |  |  |  |  |  |  |  |  |  |  |  |  |  |  |  | 13.3%; Low/weak |
| Pain Thermometer |  |  |  |  |  |  |  |  |  |  |  |  |  |  |  | 13.3%; Low/weak |
| PROMIS Pain Intensity Instrument |  |  |  |  |  |  |  |  |  |  |  |  |  |  |  | 13.3%; Low/weak |
| PROMIS v2.0 Brief Profile Sex FS |  |  |  |  |  |  |  |  |  |  |  |  |  |  |  | 13.3%; Low/weak |
| SB Clinical Factors: Spina Bifida Severity and Pain |  |  |  |  |  |  |  |  |  |  |  |  |  |  |  | 13.3%; Low/weak |
| The Kessler Psychological Distress Scale (K10) |  |  |  |  |  |  |  |  |  |  |  |  |  |  |  | 13.3%; Low/weak |
| The Utrecht Scale for Evaluation of Rehabilitation-Participation Restrictions Scale |  |  |  |  |  |  |  |  |  |  |  |  |  |  |  | 13.3%; Low/weak |
| Barth Syndrome – Symptom Assessment |  |  |  |  |  |  |  |  |  |  |  |  |  |  |  | 6.7%; Low/weak |
| Berlin Social Support Scale |  |  |  |  |  |  |  |  |  |  |  |  |  |  |  | 6.7%; Low/weak |
| Distress Thermometer |  |  |  |  |  |  |  |  |  |  |  |  |  |  |  | 6.7%; Low/weak |
| Family APGAR |  |  |  |  |  |  |  |  |  |  |  |  |  |  |  | 6.7%; Low/weak |
| General Anxiety Disorder-7 |  |  |  |  |  |  |  |  |  |  |  |  |  |  |  | 6.7%; Low/weak |
| Hopkins Symptom Checklist-25 (HSCL-25) |  |  |  |  |  |  |  |  |  |  |  |  |  |  |  | 6.7%; Low/weak |
| Hopkins Symptom Checklist-10 (HSCL-10) |  |  |  |  |  |  |  |  |  |  |  |  |  |  |  | 6.7%; Low/weak |
| Perceived health competence scale |  |  |  |  |  |  |  |  |  |  |  |  |  |  |  | 6.7%; Low/weak |
| PROMIS Pain Interference – Short Form 6a |  |  |  |  |  |  |  |  |  |  |  |  |  |  |  | 6.7%; Low/weak |
| PROMIS v2.0 Social Isolation – Short form 8a |  |  |  |  |  |  |  |  |  |  |  |  |  |  |  | 6.7%; Low/weak |
| PROMIS v2.0 - Satisfaction with social roles and activities – short form 8a |  |  |  |  |  |  |  |  |  |  |  |  |  |  |  | 6.7%; Low/weak |
| PROMIS v2.0 Ability to Participate in Social Roles and Activities – Short Form 8a |  |  |  |  |  |  |  |  |  |  |  |  |  |  |  | 6.7%; Low/weak |
| Self-Efficacy for Managing Chronic Disease |  |  |  |  |  |  |  |  |  |  |  |  |  |  |  | 6.7%; Low/weak |
| Social Network and Support Assessment Tool |  |  |  |  |  |  |  |  |  |  |  |  |  |  |  | 6.7%; Low/weak |
| State-Trait Anxiety Inventory |  |  |  |  |  |  |  |  |  |  |  |  |  |  |  | 6.7%; Low/weak |
| Subjective Happiness Scale (SHS) |  |  |  |  |  |  |  |  |  |  |  |  |  |  |  | 6.7%; Low/weak |
| The Multidimensional Scale of Perceived Social Support (MSPSSI) |  |  |  |  |  |  |  |  |  |  |  |  |  |  |  | 6.7%; Low/weak |
| The Multidimensional Scale of Perceived Social Support (MSPSSI) – Short Form |  |  |  |  |  |  |  |  |  |  |  |  |  |  |  | 6.7%; Low/weak |
